# *In vitro* neurons learn and exhibit sentience when embodied in a simulated game-world

**DOI:** 10.1016/j.neuron.2022.09.001

**Published:** 2022-12-07

**Authors:** Brett J. Kagan, Andy C. Kitchen, Nhi T. Tran, Forough Habibollahi, Moein Khajehnejad, Bradyn J. Parker, Anjali Bhat, Ben Rollo, Adeel Razi, Karl J. Friston

**Affiliations:** 1 Cortical Labs, Melbourne, Australia; 2 The Ritchie Centre, Hudson Institute of Medical Research, Clayton, VIC, Australia; 3 Department of Materials Science and Engineering, Monash University, Melbourne, VIC, Australia; 4 Wellcome Trust Centre for Neuroimaging, Institute of Neurology, University College London, London, UK; 5 Department of Neuroscience, Central Clinical School, Monash University, Melbourne, Australia; 6 Turner Institute for Brain and Mental Health, Monash University, Clayton, VIC, Australia; 7 Monash Biomedical Imaging, Monash University, Clayton, VIC, Australia; 8 CIFAR Azrieli Global Scholars Program, CIFAR, Toronto, Canada; 9 Department of Biomedical Engineering, The University of Melbourne, Parkville, Australia; 10 Department of Data Science and AI, Monash University, Melbourne, Australia

**Keywords:** cell culture, microphysiological systems, learning, intelligence, electrophysiology, neurocomputation, synthetic biological intelligence, free energy principle, in vitro, neurons

## Abstract

Integrating neurons into digital systems may enable performance infeasible with silicon alone. Here, we develop *DishBrain*, a system that harnesses the inherent adaptive computation of neurons in a structured environment. *In vitro* neural networks from human or rodent origins are integrated with *in silico* computing via a high-density multielectrode array. Through electrophysiological stimulation and recording, cultures are embedded in a simulated game-world, mimicking the arcade game “Pong.” Applying implications from the theory of active inference via the free energy principle, we find apparent learning within five minutes of real-time gameplay not observed in control conditions. Further experiments demonstrate the importance of closed-loop structured feedback in eliciting learning over time. Cultures display the ability to self-organize activity in a goal-directed manner in response to sparse sensory information about the consequences of their actions, which we term synthetic biological intelligence. Future applications may provide further insights into the cellular correlates of intelligence.

## Highlights

• Improvements in performance or “learning” over time following closed-loop feedback
• Learning observed from both human and primary mouse cortical neurons
• Systems with stimulus but no feedback show no learning
• Dynamic changes observed in neural electrophysiological activity during embodiment

## Graphical abstract

**Figure:**
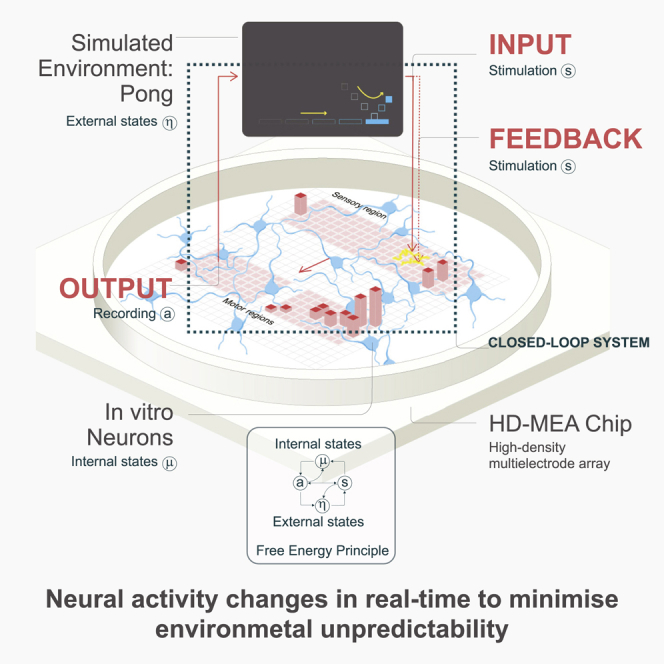

## Teaser

The *DishBrain* system is the first real-time synthetic biological intelligence platform that demonstrates that biological neurons can adjust firing activity in a way that suggests the ability to learn to perform goal-oriented tasks when provided with simple electrophysiological sensory input and feedback while embodied in a game-world.

## Introduction

**Figure 1:**
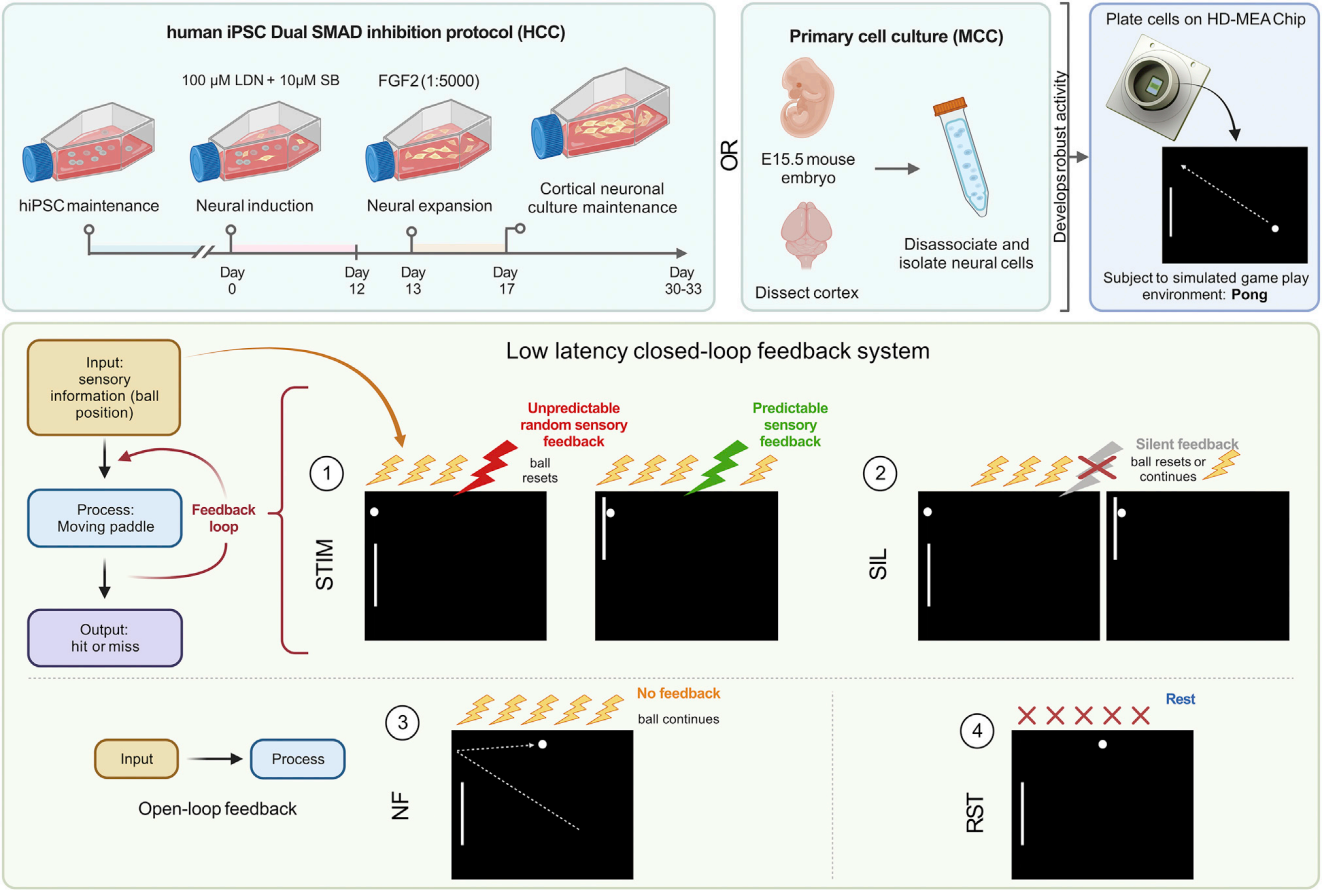
*DishBrain* system and experimental protocol schematic Neuronal cultures derived from hiPSC via DSI protocol, NGN2 lentivirus-directed differentiation, or primary cortical cells from E15.5 mouse embryos were plated onto HD-MEA chips and embedded in a stimulated game-world of “Pong” via the *DishBrain* system. Different *DishBrain* environments were created by altering the pattern of sensory information (yellow bolts), feedback (colored bolts), or no stimulus (red crosses) to demonstrate (1 and 2) low-latency, closed-loop feedback system (stimulation (STIM) and silent (SIL) treatment); (3) no-feedback (NF) system to demonstrate an open-loop feedback configuration; and (4) rest (RST) configuration to demonstrate a system in which sensory information is absent. Interactive visualizer of activity and gameplay: https://bit.ly/3DSi4Eg.

Harnessing the computational power of living neurons to create synthetic biological intelligence (SBI), previously confined to the realm of science fiction, may now be within reach of human innovation. The superiority of biological computation has been widely theorized with attempts to develop biomimetic hardware supporting neuromorphic computing (Kumar et al., 2020). Yet no artificial system outside biological neurons is capable of supporting at least third-order complexity (able to represent three state variables), which is necessary to recreate the complexity of a biological neuronal network (BNN) (Izhikevich, 2006; Kumar et al., 2020). While significant progress has been made in mapping *in vivo* neural computation, there are technical limits to exploring this *in vitro* (Barron et al., 2020). Here, we aim to establish functional *in vitro* BNNs from embryonic rodent and human-induced pluripotent stem cells (hiPSCs) on high-density multielectrode arrays (HD-MEAs) to demonstrate that these neural cultures can exhibit biological intelligence—as evidenced by learning in a simulated gameplay environment to alter activity in an otherwise arbitrary manner—in real time (Figure 1). It is proposed that these neural cultures would meet the formal definition of sentience as being “responsive to sensory impressions” through adaptive internal processes (Friston et al., 2020). Instantiating SBIs could herald a paradigm shift of research into biological intelligence, including pseudo-cognitive responses as part of drug screening (Kagan et al., 2022; Myers, 2017), bridging the divide between single-cell and population-coding approaches to understanding neurobiology (Ebitz and Hayden, 2021), exploring how BNNs compute to inform machine-learning approaches (Mattar and Lengyel, 2022), and potentially giving rise to silico-biological computational platforms that surpass the performance of existing purely silicon hardware. Theoretically, generalized SBI may arrive before artificial general intelligence (AGI) due to the inherent efficiency and evolutionary advantage of biological systems (Buchanan, 2018).

This system, termed *DishBrain*, can leverage the inherent property of neurons to share a “language” of electrical activity to link silicon and BNN systems through electrophysiological stimulation and recording. Given the compatibility of hardware and cells (wetware), it is necessary to investigate what processes would result in intelligent (goal-directed) behavior when BNNs are embodied through a closed-loop system. Two interrelated processes are required for sentient behavior in an intelligent system. Firstly, the system must learn how external states influence internal states via perception and how internal states influence external states via action. Secondly, the system must infer from its sensory states when it should adopt a particular activity and how its actions will influence the environment. To address the first imperative, custom software drivers were developed to create low-latency closed-loop feedback systems that simulated exchange with an environment for BNNs through electrical stimulation. Closed-loop systems afford an *in vitro* culture “embodiment” by providing feedback on the causal effect of the behavior from the cell culture. Embodiment requires a separation of internal versus external states where feedback of the effect of an action on a given environment is available. Previous works, both *in vitro* and *in silico*, have shown that electrophysiological closed-loop feedback systems engender significant network plasticity (Bakkum et al., 2008a; Chao et al., 2008). Further support is found *in vivo* by disrupting the closed-loop coupling between visual feedback and motor outputs in the primary visual cortex of mice (Attinger et al., 2017), highlighting the link between feedback and the development of functional behavior in BNNs.

To address the second requirement, a theoretical framework for how intelligent behavior may arise was tested by the *DishBrain* system. One proposition for how intelligent behavior may arise in an intelligent system embodied in an environment is the theory of active inference via the free energy principle (FEP) (Friston et al., 2012). The FEP suggests a testable implication that at every spatiotemporal scale, any self-organizing system separate from its environment seeks to minimize its variational free energy (VFE) (Friston, 2010; Palacios et al., 2020; Parr and Friston, 2019). The gap between the model predictions and observed sensations (“surprise” or “prediction error”) may be minimized in two ways: by optimizing probabilistic beliefs about the environment to make predictions more like sensations or by *acting* upon the environment to make sensations conform to its predictions. This model then implies a common objective function for action and perception that scores the fit between an internal model and the external environment. Under this theory, BNNs hold “beliefs” about the state of the world, where learning involves updating these beliefs to minimize their VFE or actively change the world to make it less surprising (Parr and Friston, 2018, 2019). If true, this implies that it should be possible to shape BNN behavior by simply presenting unpredictable feedback following “incorrect” behavior. Theoretically, BNNs should adopt actions that avoid the states that result in unpredictable input. By developing a system that allows for neural cultures to be embodied in a simulated game-world, we are not only able to test whether these cells are capable of engaging in goal-directed learning in a dynamic environment, but we are also able to investigate the foundations of intelligence.

Previous work supports that *in vitro* neuronal networks can perform blind-source separation in an open-loop environment via state-dependent Hebbian plasticity consistent with the FEP (Isomura et al., 2015; Isomura and Friston, 2018). We sought to build upon this work to test the theory of active inference, which applies the FEP to sentient systems that not only adapt to fit their environment, but also act upon their environment to fit it to themselves. We therefore hypothesize that when provided a structured external stimulation simulating the classic arcade game “Pong” within the *DishBrain* system, the BNN would modify internal activity to avoid adopting states linked to unpredictable external stimulation. This minimization of input unpredictability would manifest as the goal-directed control of the simulated “paddle” in this simplified simulated “Pong” environment.

## Results

### Growth of neuronal “wetware” for computation

**Figure 2:**
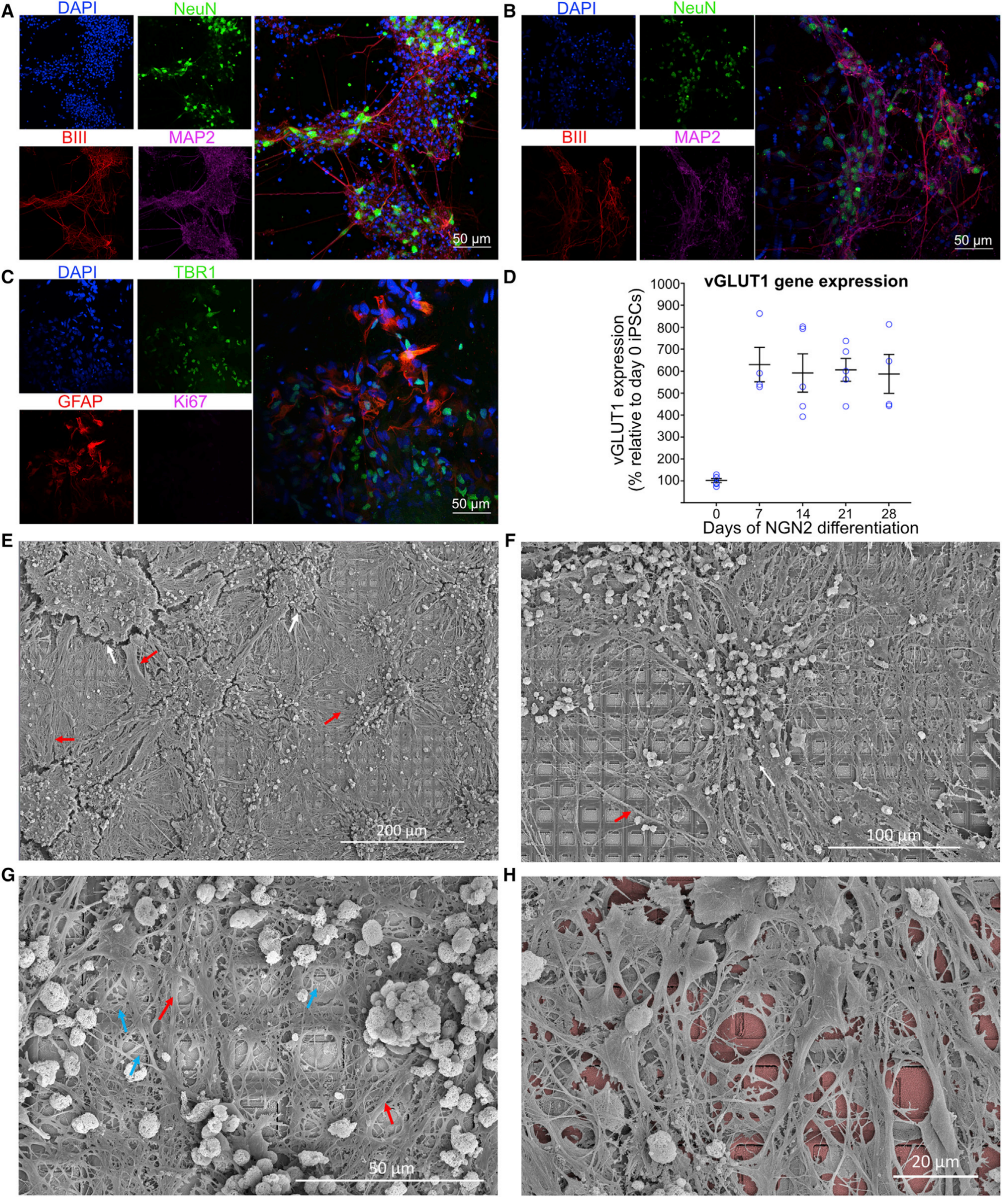
Cortical cells form dense interconnected networks (A and B) Cortical cells from E15 mouse brains and differentiated from hiPSCs, respectively. DAPI in blue stains all cells, NeuN in green shows neurons, beta III tubulin (BIII) marks axons, while MAP2 marks dendrites. Scale bar = 50μm. (C) GFAP shows supporting astrocytes, critical for long-term functioning; TBR1 marks cortex-specific cells. No Ki67, a marker of dividing cells, was observed with these cultures. Scale bar = 50μm. (D) Gene expression studies over 28 days demonstrated increased expression of the glutamatergic neural marker, vesicular glutamate transporter 1 (vGLUT1). (E–G) Neurons differentiated from hiPSCs using the DSI protocol, maintained on MEA for >3 months. White arrows show regions of shrinkage within the cultures, red arrows show bundles of axons, and blue arrows show single neurite extensions. Note the dense coverage over the HD-MEA and overlapping connections extended from neuronal soma present in all cultures across multiple electrodes. Scale bars: E = 200μm, F = 100μm, G = 50μm (H) Has false coloring to highlight the HD-MEA electrodes beneath the cells. Scale bar = 20μm.

Cortical cells from the dissected cortices of rodent embryos can be grown on MEAs in nutrient-rich media and maintained for months (Bardy et al., 2015; Lossi and Merighi, 2018). These cultures will develop complicated morphology with numerous dendritic and axonal connections, leading to functional BNNs (Kamioka et al., 1996; Wagenaar et al., 2006). Primary neural cultures from embryonic day 15.5 (E15.5) mouse embryos were cultured, with representative cultures shown in Figure 2A. HiPSCs were differentiated into monolayers of active heterogeneous cortical neurons, which have been shown to display mature functional properties (Denham et al., 2012; Denham and Dottori, 2009; Shi et al., 2012). Using dual SMAD inhibition (DSI) (Denham et al., 2012; Fattahi et al., 2015), we developed long-term cortical neurons that formed dense connections with supporting glial cells (Figures 2B and 2C). Finally, we aimed to expand our study using a different method of hiPSC differentiation—NGN2 direct reprogramming (Pak et al., 2018; Zhang et al., 2013)—used in our final part of this study investigating feedback mechanisms. This high-yield method resulted in cells displaying pan-neuronal markers (Figures S1A and S1B). These cells typically display a high proportion of excitatory glutamatergic cells, quantified using qPCR, shown in Figure 2D. Integration of these neuronal cultures on the HD-MEAs was confirmed via scanning electron microscopy (SEM) on cells that had been maintained for >3 months (Figure 2E). Densely interconnected dendritic networks could be observed in neuronal cultures forming interlaced networks spanning the MEA area (Figure 2F). These neuronal cultures appeared to rarely follow the topography of the MEA, being more likely to form large clusters of connected cells with dense dendritic networks (Figures 2G and 2H). This is likely due to the large size of an individual electrode within the MEA and potentially also chemotactic effects that can contribute to counteract the effect of substrate topography on neurite projections (Mattotti et al., 2012).

### Neural cells show well-characterized spontaneous action potentials that develop over time

**Figure 3:**
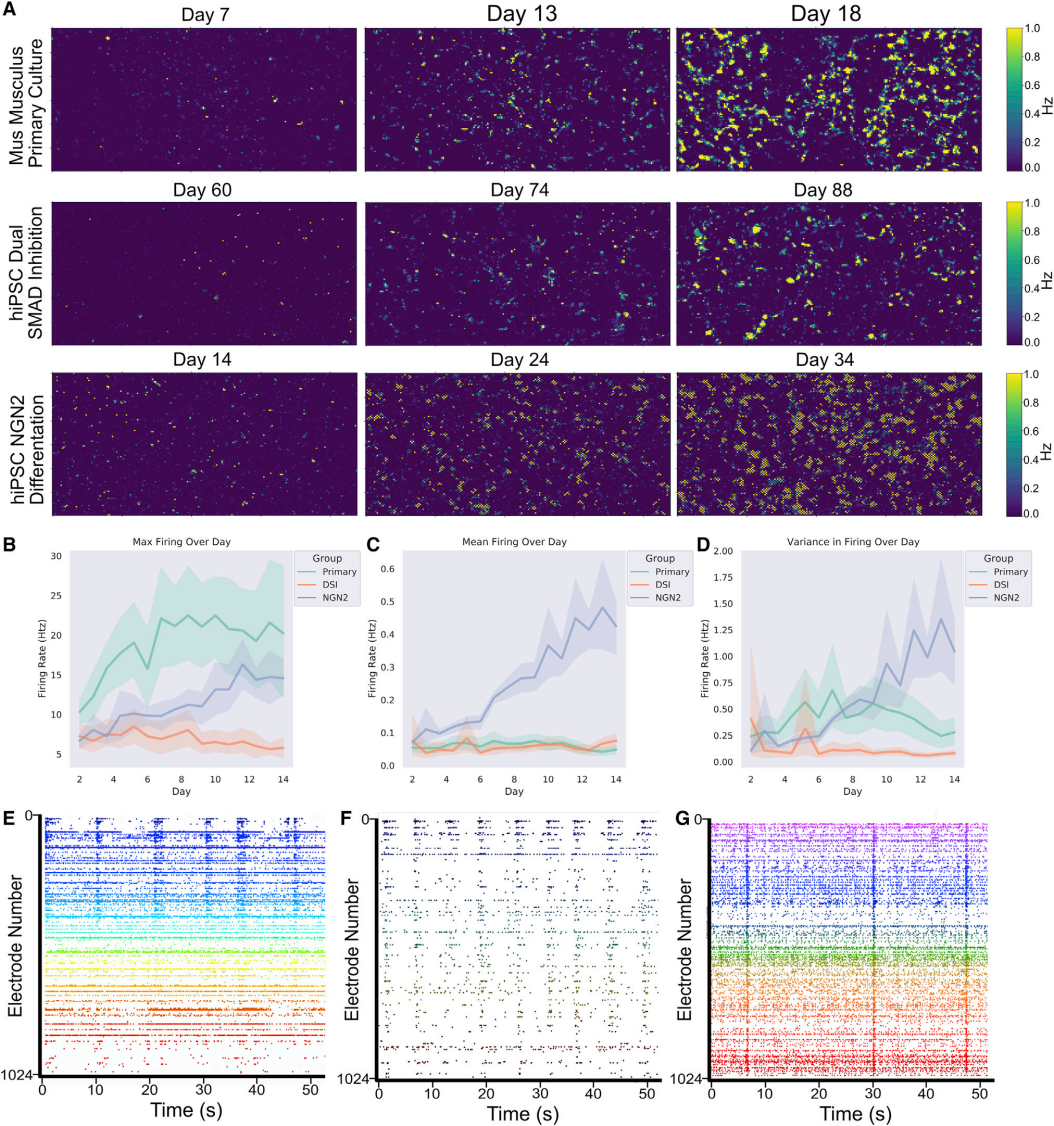
Cortical cells display spontaneous electrophysiological activity Shaded error = 95% confidence intervals. (A) Firing rate for E15.5 primary rodent cortical cells, hiPSC cells differentiated to cortical neurons via DSI, and hiPSC cells differentiated via NGN2 direct differentiation. Note different time points for each cell type. Scale bar displays firing frequency (Hz) from 0.0 to 1.0. (B) Max firing was consistently different between cortical cells from a primary source and cortical cells differentiated from hiPSCs. (C and D) Mean activity between hiPSCs differentiated using DSI and primary cortical cultures was generally similar, while hiPSCs differentiated using the NGN2 method continued to increase. This is reflected in (D), where the former two cell types displayed minimal changes in the variance in firing within a culture, while the latter increased variance over time. (E, F, and G) Showcases raster plots over 50 s, where each dot is a neuron firing an action potential colored to help distinguish channel firing and stars indicate time points with observed bursting activity. Note the differences between mid-stage cortical cells from a DIV14 primary rodent culture (E) compared with more mature DIV73 human cortical cells (F) differentiated from iPSCs using the DSI and NGN2 direct differentiated neurons (G) approach described in text, in terms of synchronized activity and stable firing patterns. While all display synchronized activity, there is a difference in the overall levels of activity represented in (B–D).

*In vitro* development of electrophysiological activity in neural systems at high spatial and temporal resolution was mapped. Robust activity in primary cortical cells from E15.5 rodents was found at days *in vitro* (DIV) 14 (Figures 3A and 3E) where bursts of synchronized activity were regularly observed, as previously demonstrated (Kamioka et al., 1996; Wagenaar et al., 2006). In contrast, similar to previous reports (Shi et al., 2012), synchronized bursting activity was not observed in cortical cells from an hiPSC background differentiated using DSI until DIV 73 (Figures 3A and 3F). HiPSCs differentiated using NGN2 direct reprogramming showed activity much earlier, typically between days 14 and 24 (Figures 3A and 3G). Electrophysiological maturation was monitored with daily activity scans. While max firing rate typically increased and remained relatively stable over time for all cell types during the testing period (Figure 3B), changes were observed in both the mean firing rate (Figure 3C) and variance in firing rate (Figure 3D) over the days of testing; in particular, hiPSCs differentiated using the NGN2 direct reprogramming method showed a considerable increase in mean firing rate and the variance in firing over days of testing.

### Building a modular, real-time platform to harness neuronal computation

**Figure 4:**
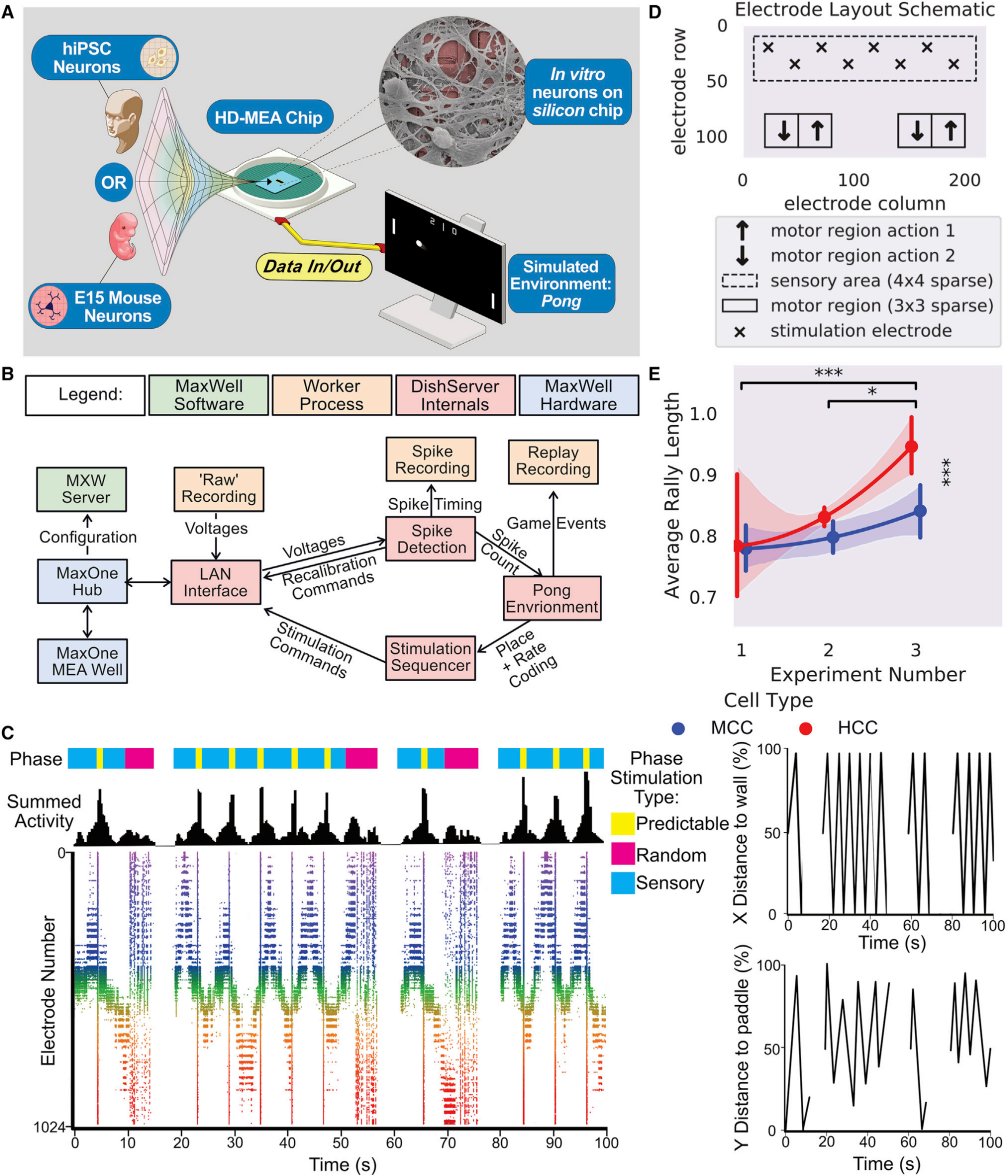
Schematics and pilot testing with increasing informational density (A) Diagrammatic overview of *DishBrain* setup. (B) Software components and data flow in the *DishBrain* closed-loop system. Voltage samples flow from the MEA to the “Pong” environment, and sensory information flows back to the MEA, forming a closed loop. Full caption in Figure S2. (C) Schematic showing the different phases of stimulation to the culture. In line with this is the corresponding summed activity on the raster plot over 100 seconds. The appearance of random stimulation after a ball missing versus system-wide predictable stimulation upon a successful hit is apparent across all three representations. Corresponding images on the right show the position of the ball on both x and y axis relative to the paddle and back wall in percentage of total distance shown on the same timescale. (D) Final electrode layout schematic for *DishBrain* Pong-world gameplay. (E) ^∗^ = p < 0.05, ^∗∗∗^ = p < 0.001; error bars = 95% CI. Shows average rally length over three distinct experiment rounds during design of *DishBrain* Pong-world where each subsequent experiment provided higher density information on ball position than the previous. MCC tested over 272 sessions, n = 50 biological replicates; HCC tested over 579 sessions, n = 18 biological replicates.

The *DishBrain* system was developed to leverage neuronal computation and interact with neurons embodied in a simulated environment (STAR Methods; Figure 4A; Video S2). The *DishBrain* environment is a low-latency, real-time system that interacts with the vendor MaxOne software, allowing it to be used in ways that extend its original functions (Figure 4B). This system can record electrical activity in a neuronal culture and provide “sensory” (non-invasive) electrical stimulation comparably to the generation of action potentials by activity in the neuronal network (Ruaro et al., 2005). Using the coding schemes described in STAR Methods, external electrical stimulations convey a range of information. For our purposes, we opted for three distinct information categories: predictable, random, and sensory (STAR Methods, Figure 4C). *DishBrain* (Figure S2) was designed to integrate these functions to “read” information from and “write” sensory data to a neural culture in a closed-loop system so neural “action” influences future incoming “sensory” stimulation in real time. The intent was to embody BNNs in a virtual environment and to quantify demonstrable learning effects.

**Figure 5:**
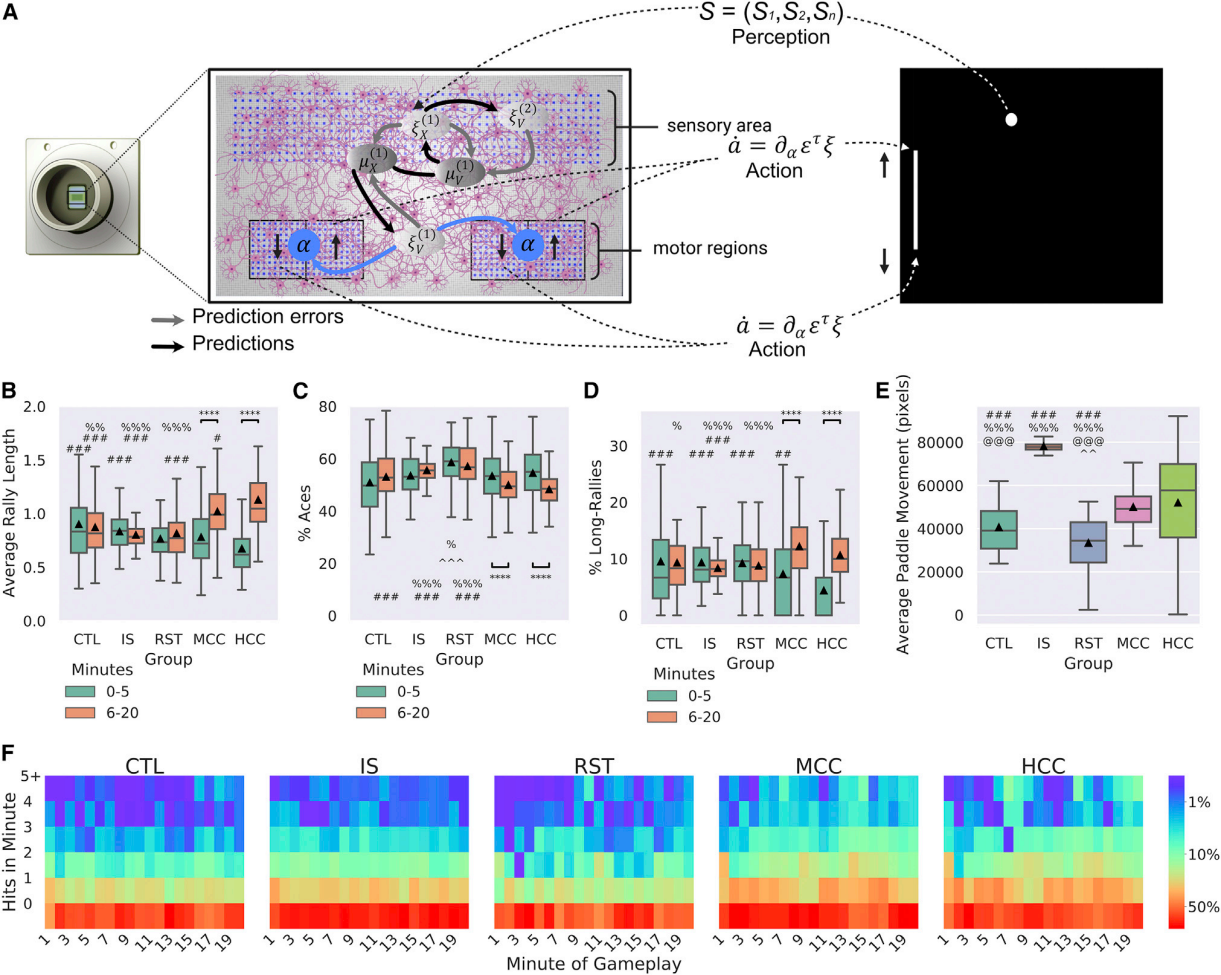
Embodied cortical neurons show significantly improved performance in “Pong” when embodied in a virtual game-world 399 test-sessions were analyzed with biological replicates: 80-CTL (n = 6), 42-RST (n = 20), 38-IS (n = 3), 101-MCCs (n = 9), 138-HCCs (n = 11). Significance bars show within-group differences denoted with ^∗^. Symbols show between-group differences at the given timepoint: # = versus HCC; % = versus MCC; ˆˆ = versus CTL; @ = versus IS. The number of symbols denotes the p value cutoff, where 1 = p < 0.05, 2 = p < 0.01, 3 = p < 0.001, and 4 = p < 0.0001. Boxplots show interquartile range, with bars demonstrating 1.5× interquartile range, the line marks the median, and ▲ marks the mean. (A) Schematic of how neurons may engage in the game-world under active inference denoting a gradient flow on variational free energy, expressed in terms of neural activity minimizing prediction errors. ε is prediction error, ξ represents a precision-weighted prediction error. Precision can be regarded as a Kalman gain in Kalman filtering; ‘a’ corresponds to action. (B–D) Experimental groups according to time point 1 (T1; 0–5 min) and time point 2 (T2; 6–20 min). (B) Average performance between groups over time, where only experimental (MCC: *t* = 6.15, p = 5.27^−08^ and HCC: *t* = 10.44, p = 3.92^−19^) showed significant improvement and higher average rally length against all control groups at T2. (C) Average number of aces between groups and over time, only MCC (*t* = 2.67, p = 0.008) and HCC (*t* = 5.95, p = 2.13^−08^) differed significantly over time. The RST group had significantly more aces compared with the CTL, IS, MCC, and HCC groups at T1 and compared with the CTL, MCC, and HCC at T2. Only MCCs and HCCs showed significant decreases in the number of aces over time, indicating learning. At T2 they also showed fewer aces compared with the IS group, but only the HCC group was significantly less than CTL. (D) Average number of long rallies (>3) performed in a session. At T1, the HCC group had significantly fewer long rallies compared with all control groups (CTL, IS, and RST). However, both the MCC (*t* = 5.55, p = 2.36^−07^) and HCC (*t* = 10.38, p = 5.27^−19^) groups showed significantly more long rallies over time. By T2, the HCC group displayed significantly more long rallies compared with the IS group. The HCC group also displayed significantly more long rallies compared with all CTL, IS, and RST control groups. (E) The average distance that the paddle moved during a session was found to have no obvious relationship with average rally length as the IS control groups showed a higher movement than the experimental groups, while CTL and RST were lower. As such, the observed learning effects are not likely due to stimulation, leading to increased activity of paddle movement. (F) Distribution of frequency of mean summed hits per minute among groups shows obvious differences; scale bar shows the probability the number of hits in the given minute under that condition.

Video S2. Representative movie of interactive SpikeStream visualizer and overview of system setup, related to Figure 5
Representative movie of a paddle being controlled by the activity of living neurons to play a simulated game of “Pong” in the *SpikeStream* interactive visualizer with associated descriptions of the methods and summary of key results. This is also available live in real time from any active culture in the *DishBrain* system

The initial proof of principle using *DishBrain* was to simulate the classic arcade game “Pong” by delivering inputs to a predefined sensory area of 8 electrodes (Figure 4D). Electrodes were arranged in a manner that would allow a coarse, yet topographically consistent, place coding, consistent with *in vivo* systems (see STAR Methods) (Baranes et al., 2012; Patel et al., 2014; Shlens et al., 2006). The electrophysiological activity of defined motor regions was gathered—in real time—to move a paddle. If this activity did not result in an interception of the ball by the paddle, an unpredictable stimulus was delivered (150mV voltage at 5Hz for 4 seconds; see STAR Methods), after which time the ball stimulation would recommence on a random vector. In contrast, if a successful interception occurred, a predictable stimulus was delivered across all electrodes simultaneously at 100Hz for 100ms (briefly interrupting the regular sensory stimulation) before the game continued predictably. Preliminary investigations compared different motor region configurations to verify that motor region setup did not introduce bias (paddle movement that aligned to the ball position) from input stimulation alone (STAR Methods; Figure S3). Experimental cultures of cortical cells showed a higher hit-miss ratio, which we defined as the average rally length, on counterbalanced split-motor configurations (Figure 4D), where media-only-filled MEAs used as a control group also showed minimal bias. Distinct areas were defined as “motor regions,” where activity in motor region action 1 moved the paddle “up” and activity in motor region action 2 moved the paddle “down.” This fixed layout means that monolayers of cells—with a random distribution that is arbitrary in relation to the “motor” configuration—will need to adopt distinct firing patterns through self-organization (and raises the question to what extent this self-organization will occur).

### Increasing the density of sensory information input leads to increased performance

The *DishBrain* protocol was refined over three pilot studies, each increasing the density of sensory information. Pilot study 1 operated with a 4Hz stimulation that only involved place coding, where the location of the stimulation corresponded to the position of the ball on the y axis. Pilot study 2 investigated different configurations and introduced activity-based weighting to motor regions to account for cell density or activity differences. Pilot study 3 adopted the layout in Figure 4D and changed to the combined rate (4–40Hz) and place-coding method of data input. This combined rate and place coding has compelling biological similarities conceptually to the rodent barrel cortex, suggesting this encoding is physiologically coherent (Harrell et al., 2020; Ly et al., 2012; Petersen et al., 2001). Gameplay for the final fifteen minutes for each culture type was compared (Figure 4E and Table S1). Cultures displayed a significant increase in the average rally length between the second and final pilot studies and the first and final pilot studies. Between cultures, human cortical cells (HCCs) had significantly longer average rally lengths than cultures with mice cortical cells (MCCs) (Table S2). Overall, these results support that increasing the amount of sensory information improved performance, even when cell culture features were kept constant.

### BNNs learn over time when embodied in a gameplay environment

To test the predictions of the FEP (Figure 5A) using selected parameters (STAR Methods), cortical cells (MCCs and HCCs) were compared with media-only controls (CTL); rest sessions (RST), where active cultures controlled the paddle but received no sensory information; and in-silico (IS) controls that mimicked all aspects of the gameplay except the paddle were driven by random noise over 399 test sessions (80-CTL [n = 6 MEA], 42-RST [n = 20 cultures], 38-IS [n = 3 seeds], 101-MCCs [n = 9 cultures], 138-HCCs [n = 11 cultures]). The average rally length showed a significant interaction (Figure 5B and Table S1) between group and time (first 5 and last 15 min). Only the MCC and HCC cultures showed evidence of learning with significantly increased rally lengths over time. Further, it was found that during gameplay in timepoint 1 (T1), key significant differences were observed (Table S1): the HCC group performed significantly worse than MCC, CTL, and IS groups (Table S2). This suggests that HCCs perform worse than controls when first embodied in an environment, suggesting an initial maladaptive control of the paddle or perhaps an exploratory behavior. Notably, at timepoint 2 (T2), this trend was reversed; the MCC and HCC groups significantly outperformed all control groups along with HCC showing a slight but significant outperformance over the MCC group (Tables S1 and S2). This data demonstrates a significant learning effect in both experimental groups absent in the control groups, along with evidence that the learning capabilities differ between mice and human cells in line with previous results (Video S1).

Video S1. Representative movie of DishBrain system in action, related to Figure 1
Representative video of a paddle being controlled by the activity of living neurons to play a simulated game of “Pong.” It is of particular interest to note how frequently after a successful hit the paddle leads where the ball will eventually end up on the return, even before the ball hits the backwall

### Learning effects in BNNs are observed across additional measures

Other key gameplay characteristics, such as the number of times the paddle failed to intercept the ball without a single hit defined as “aces,” and the number of gameplays with greater than 3 consecutive hits defined as “long rallies,” were calculated. As with average rally length, significant interactions between groups and time were found for aces and long rallies (Table S1). Only the MCC and HCC groups showed significantly fewer aces in T2 compared with T1 (Figure 5C and Table S2). Likewise, only the MCC and HCC groups showed significantly more long rallies in T2 compared with the first (Figure 5D and Table S2). Collectively, the data shows that both experimental cultures (HCCs and MCCs) improved performance by reducing how often they missed the initial serve and achieving more consecutive hits or longer rallies.

Differences between groups at T1 were found both for aces and long rallies (Table S1). The RST condition displayed significantly more aces than the CTL and MCC groups (Table S2), suggesting a degree of sporadic behavior that the cells exhibit when initially introduced to the rest period from gameplay that results in this behavior. When the number of long rallies at T1 was investigated, it was found that only HCCs had significantly fewer long rallies (Table S2). This finding complements the reduced average rally lengths discussed above. Significant differences between groups at T2 were also found for aces and long rallies (Figures 5C and 5D and Table S1). Notably, the HCC group showed significantly fewer aces than CTL, RST, and IS groups (Table S1). The MCC group also showed significantly fewer aces than RST and IS groups, but not the CTL group (Table S2). In contrast, for long rallies, the MCC group showed significantly more than the CTL, RST, and IS groups (Table S2), yet the HCC group only showed significantly more long rallies compared with the IS group, but not RST or CTL (Table S2).

No learning effect was found in electrically inactive non-neural cells (HEK293T cells) and media-only controls (Figures S4A–S4C). Further, a significant negative correlation between percentage of aces and percentage of long rallies of both MCCs and HCCs was found, suggesting that the performance was not arising from maladaptive behavior such as fixing the paddle to a single corner (Figure S4D). Whether stimulation alone may cause greater movement of the paddle and that this may result in the observed learning effects was also investigated. As Figure 5E shows, while there were significant differences observed in paddle movement between conditions (Table S1), for the CTL and RST, this resulted in significantly lower movement relative to the other groups, with the RST being the lowest movement of all groups (Table S2). The IS control group showed significantly more paddle movement than all other groups yet displayed no meaningfully different performance metrics to the other control groups (CTL and RST) (Table S2). Additionally, Figure S4E shows no significant correlation between paddle movement and average rally length was observed, supporting that movement alone of the paddle does not explain the observed learning effects. Wholistically, Figure 5F emphasizes that both MCCs and HCCs showed fewer aces and more long rallies in T2 compared with T1, reiterating the observed learning effect over time. This can also be seen in linear regressions (Figure S4F), where only the MCC and HCC groups showed a statistically significant positive relationship between average rally length and duration of gameplay.

### BNNs require feedback for learning

**Figure 6:**
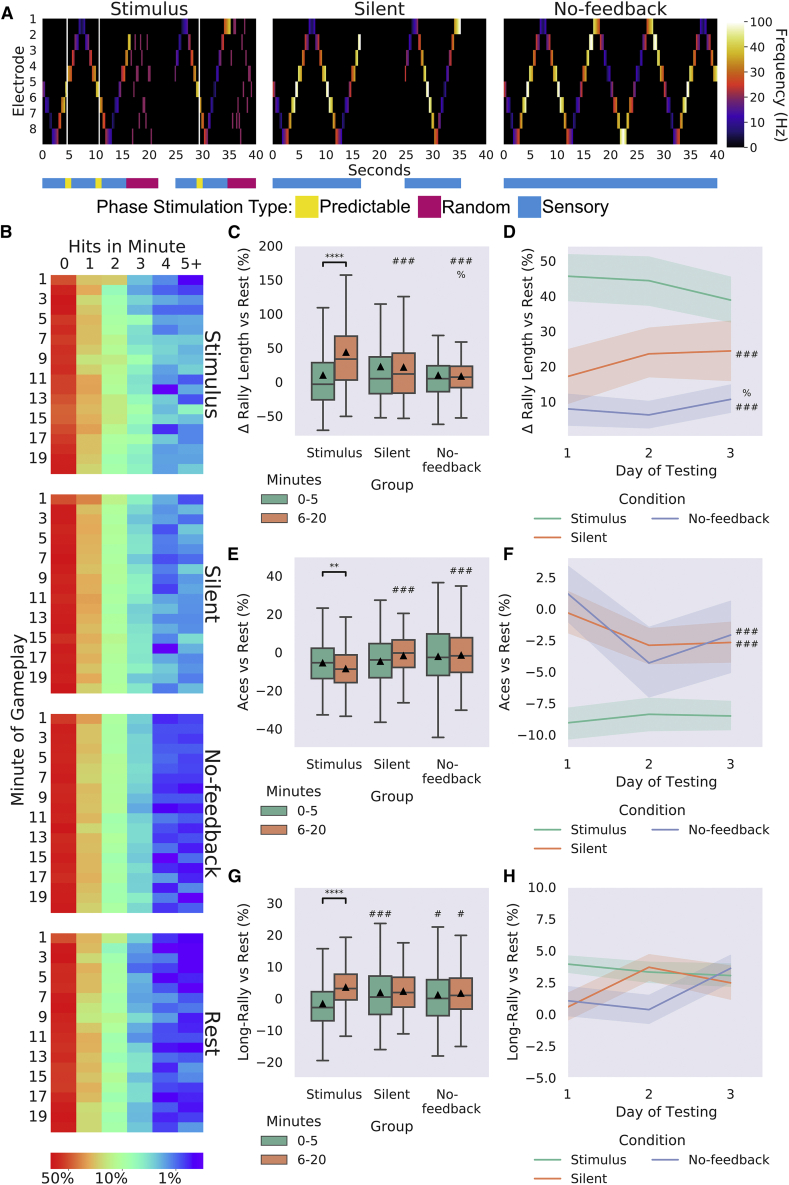
The importance of feedback in learning 486 sessions were analyzed. Significance bars show within-group differences denoted with ^∗^. Symbols show between-group differences at the given timepoint: # = versus Stimulus; % = versus Silent. The number of symbols denotes the p value cutoff, where 1 = p < 0.05, 2 = p < 0.01, 3 = p < 0.001, and 4 = p < 0.0001. Box plots show interquartile range, with bars demonstrating 1.5× interquartile range, the line marks the median, and ▲ marks the mean. Errors bands = 1 SE. (A) Schematic showing the stimulation from the 8 sensory electrodes across 40 s of the same gameplay for each of the three conditions. The bar below color codes what phase of stimulation is being delivered, where random stimulation follows a miss and predictable stimulation follows a hit in the Stimulus condition. Note the corresponding absence of any stimulation in the Silent condition and the lack of any change in sensory stimulation in the No-feedback condition. (B) Displays the probability of a certain number of hits occurring in a group at a specific minute. (C) Using different feedback schedules, the Stimulus feedback condition showed significant learning (as in Figure 5A; *t* = 7.48, p = 1.58^−12^) and outperformed Silent and No-feedback average rally length. Silent feedback also showed higher performance compared with these groups at T2. (D) Displays difference seen in (C) across day. (E) Shows similar differences versus rest performance for aces across conditions, where the Stimulus group showed significantly fewer aces across time (*t* = 3.21, p = 0.002). (F) Displays data from (E) across day. (G and H) Shows that the Stimulus condition showed significant increase (*t* = 3.21, p = 0.002) across timepoints; however, as in (H), no differences were found across time for long rallies.

To investigate the importance of the feedback type for learning, cultures, both MCCs and HCCs, were tested under 3 conditions for 3 days, with 3 sessions per day resulting in a total of 486 sessions. Condition 1 (Stimulus; n = 27) mimicked that used above, where predictable and unpredictable stimuli were administered when the cultures behaved desirably or not, respectively. Condition 2 (Silent; n = 17) involved the stimulus feedback being replaced with a matching time period in which all stimulation was withheld, after which the game restarted with the ball beginning in a random direction. Condition 3 (No feedback; n = 15) removed the restart after a miss. When the paddle did not successfully intercept the ball, the ball would bounce and continue without interruption; the stimulus reporting ball position was still provided. The difference between these conditions is illustrated in Figure 6A. Rest-period activity was also gathered and used to normalize performance per session basis to account for differences in unstimulated activity (Figure 1).

Stimulus and Silent conditions showed an overall higher average rally length compared with Rest and No-feedback conditions (Figure 6B). When testing for differences between groups in the percentage increase of average rally length over matched rest controls, a significant interaction was found (Figure 6C and Table S1). Only the Stimulus condition showed a significant increase in average rally length over time. While no differences were found for T1, a significant main effect of group was found at T2, where the Stimulus condition had a significantly higher average rally length than the Silent and No-feedback conditions (Table S2). Interestingly, the Silent condition also significantly outperformed the No-feedback conditions, although with a smaller effect size (Table S2). Importantly, this demonstrates that information alone is insufficient; feedback is required to form a closed-loop learning system. When followed up at the level of day for T2 (Figure 6D), no significant differences over time were observed, but the same between-group differences as above were observed. This trend was similar when looking at aces both summed (Figure 6E) and across days of testing (Figure 6F). The Stimulus group at T1 showed significantly fewer long rallies compared with the Silent and No-feedback condition, being reversed at T2 with the Stimulus group showing significantly more long rallies compared with the No-feedback condition (Figure 6G). No difference was found when this was followed up across days (Figure 6H). Collectively, these results suggest that adaptive behavior seen in BNNs altering electrophysiological activity can be an emergent property of engaging with—and implicitly modelling—the environment.

### Dynamics in electrophysiological activity display coherent connectivity

**Figure 7:**
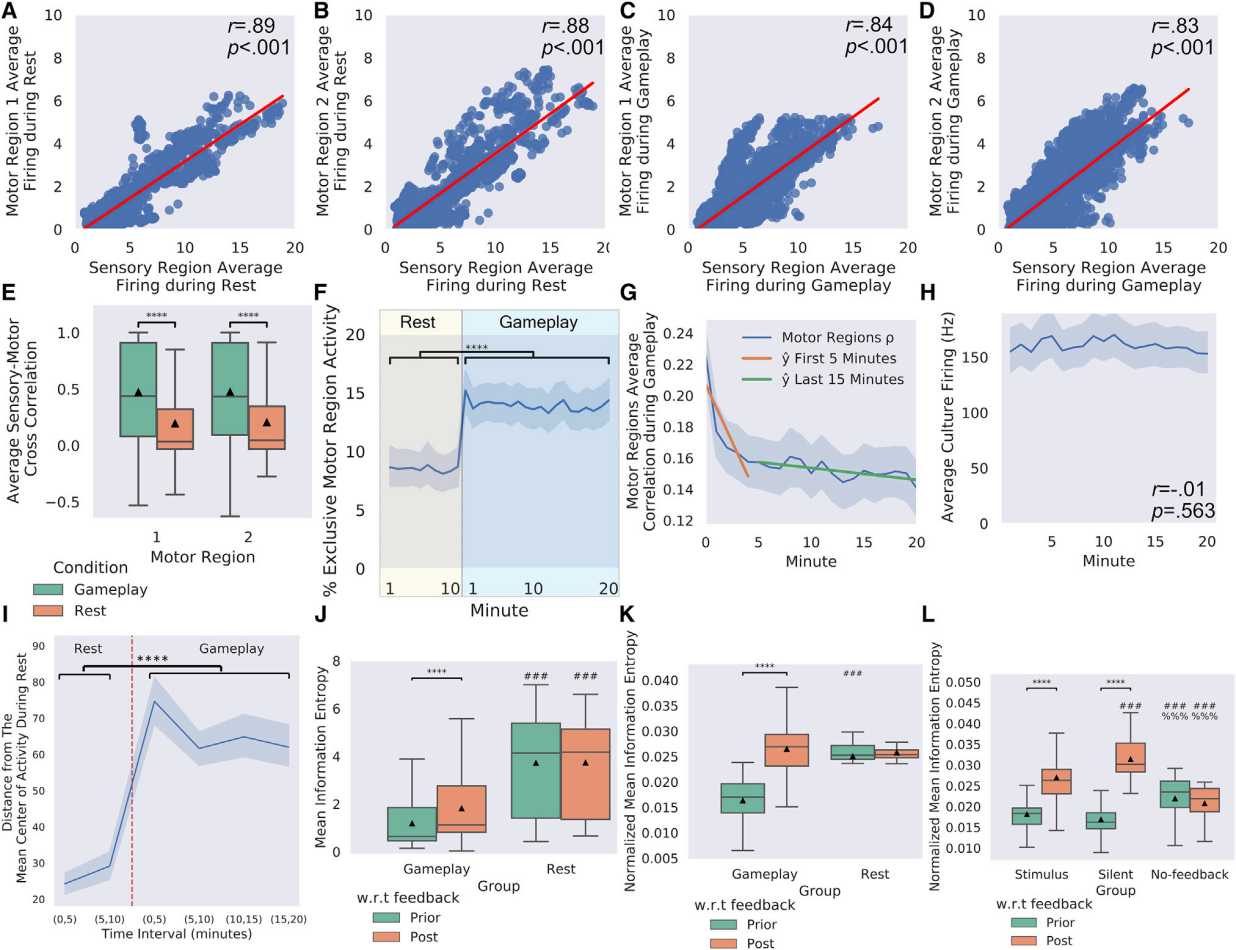
Electrophysiological activity during Gameplay and Rest 579 sessions (358 Gameplay, 221 Rest) were analyzed with n = 43 biological replicates. Significance bars show within-group differences denoted with ∗. Symbols show between-group differences at the given timepoint: # = versus Gameplay or Stimulus; % = versus Silent. The number of symbols denotes the p-value cutoff, where 1 = p < 0.05, 2 = p <0.01, 3 = p < 0.001, and 4 = p <0.0001. Box plots show interquartile range, with bars demonstrating 1.5× interquartile range, the line marks the median, and ▲marks the mean. Error bands = 1 SE. (A–D) A significant positive correlation between mean firing and performance was found between motor region 1 and 2 with the Sensory area both during Rest (A and B) and Gameplay (C and D). (E) The average cross-sensory motor correlation was significantly less during Rest, both for motor region 1 (*t* = 30.40, p = 6.61^−194^) and motor region 2 (*t* = 29.76, p = 2.76^−186^) than during Gameplay. (F) The percentage of mutually exclusive activity events per second across motor regions was calculated and found to increase significantly during Gameplay versus Rest (*t* = 14.64, p = 5.68^−48^). (G) The correlation between the two motor regions showed substantial changes over time (blue). Linear regression conducted on the first 5 min of Gameplay (orange) showed a significant negative relationship between variables that was absent in the final 15 min (teal). (H) Activity over time showed no significant changes while engaged in Gameplay (*r* = −0.01, p = 0.563), supporting that any observed learning effects over time were not related to merely gross changes in activity levels across the cultures over time. (I) Functional plasticity was assessed across cultures when engaged in Gameplay versus Rest, with a significant increase in functional plasticity found during gameplay. (J) Following random stimulation feedback, there was a significant increase in the mean information entropy during Gameplay (*t* = 4.890, p = 2.024^−6^), yet the corresponding time during Rest showed no change (*t* = 0.016, p = 0.987). Mean information entropy was lower at both pre- (*t* = 9.781, p = 3.882^−19^) and post- (*t* = 5.915, p = 1.178^−8^) feedback during Gameplay than at Rest. (K) For normalized mean information entropy, the difference relative to feedback period was increased during Gameplay (*t* = 19.337, p = 3.476^−48^), yet still no difference was observed during Rest where no feedback was delivered (*t* = 1.022, p = 0.316). Normalized mean information entropy was lower at pre- (*t* = 10.192, p = 2.139^−20^), but not post- (*t* = 0.671, p = 0.503) feedback, during Gameplay compared with Rest. (L) Feedback-related changes in normalized mean information entropy were assessed for the investigation of different feedback mechanisms. Increases following random feedback for the Stimulus condition were replicated (*t* = 9.623, p = 7.887^−19^); it was also found that the system displayed increased activity-related scores under the Silent condition feedback (*t* = 21.538, p = 7.019^−47^). The No-feedback condition showed no change in normalized mean information entropy at matched times after Bonferroni corrections (*t* = 10.192, p = 0.030). Post-hoc follow-up tests found no differences between Stimulus and Silent conditions during gameplay; both were significantly lower than for the No-feedback condition. After feedback, the Stimulus and Silent conditions were significantly higher than the No-feedback condition, with the Silent condition significantly higher than the Stimulus condition.

Electrophysiological activity during gameplay was analyzed from cultures subjected to the stimulus condition to determine functional connectivity (Mohseni Ahooyi et al., 2018). The cross correlations of firing in 100ms-time bins revealed significant, strong positive correlations between activity in the sensory region and both motor regions during Rest and Gameplay (Figures 7A–7D). However, when these correlations were calculated per bin and averaged, significantly stronger correlations were observed when cultures were in the Gameplay phase than at Rest (Figure 7E). This higher degree of connectivity would be expected if activity in the sensory region during gameplay was directly related to activity in motor regions through dynamic self-organization at the system-wide level. In line with this, when the quantity of exclusive motor region activity was calculated per second—looking for events where above-noise-level activity occurred in either motor region 1 or motor region 2, yet not both simultaneously—a significant increase in these events was found when cultures were engaged in gameplay versus rest (Figure 7F). This type of internal modulation is coherent with the observed performance of these cultures; exclusive activity changes among motor regions would be required for adaptive gameplay. Finally, to further support these results, the correlation between the two motor regions was found to vary substantially over time (Figure 7G). A linear regression of the correlation in 100ms-time bins between motor regions was found to decrease with time significantly until approximately 5 min of gameplay (*R*^2^ = 0.013, *F*(1, 2049) = 27.51, p = 1.72^−7^, *β* = −1.18, p < 0.001). After this point, little further change was observed (*R*^2^ = 0.00, *F*(1, 5181) = 2.19, p = 0.139, *β* = −0.55, p = 0.139), suggesting a degree of homeostasis. These differences do not affect the overall average culture firing that remains stable throughout the gameplay session (Figure 7H).

As electrical stimulation of neural tissue has been shown to modify neuronal activity (Bakkum et al., 2008a, 2008b; Chao et al., 2008), the functional plasticity of cultures during Gameplay was assessed compared with when at Rest as described in STAR Methods. Figure 7I suggests that closed-loop training during Gameplay displays significantly increased plasticity compared with baseline plasticity measured at Rest before training, indicating that functional plasticity was upregulated during gameplay (Table S1). To test whether learning reflects a reduction in VFE within BNNs, we used the information entropy of neuronal responses as a proxy for the average surprise (a.k.a. self-information), which is upper-bounded by VFE (see STAR Methods). We predicted a reduction in information entropy during the learning of gameplay. We further predicted an increase in entropy following unpredictable (random) feedback, reflecting and ensuing state of “surprise” (and, implicitly, high VFE), relative to pre-feedback states. For the studies reported in Figure 5, the mean information entropy was found to be lower during Gameplay than during Rest, both before and after the unpredictable feedback stimulation (Figure 7J and Table S1). There was a significant increase in mean information entropy found post-feedback relative to pre-feedback timepoints during Gameplay, but not in the corresponding timepoints during Rest where no feedback occurred. As the change in entropy can depend on the level of sensory activity pre-feedback, we normalized the mean information entropy by the number of spikes. The relationship was conserved (Figure 7K and Table S1), where a significant increase in normalized mean entropy was observed during Gameplay, but not at the corresponding timepoint during Rest where no stimulation occurred. In short, as predicted theoretically, gameplay reduced information entropy during predictable exchanges with the environment, while unpredictable feedback increased entropy during gameplay.

We repeated this analysis on the follow-up study of different feedback mechanisms reported in Figure 6. While it is important to note that the internal information entropy of the culture is not necessarily and directly tied to the external (i.e., sensory) information entropy of the stimulus being applied into a culture, it is interesting to see how cultures respond to different feedback protocols. As shown in Figure 7L, the change during the stimulus condition between the normalized mean information entropy was replicated for the standard Stimulus condition (Table S1). Of interest is the finding that during the Silent condition, the neural cultures had a higher normalized mean information entropy than even the stimulus condition post-feedback. However, the No-feedback condition showed no change relative to the period when feedback would have been applied, with a significantly higher normalized mean information entropy score than either of the other two conditions pre-feedback, yet a significantly lower score post-feedback (Table S2).

### Electrophysiological activity is linked with higher average rally length

**Figure 8:**
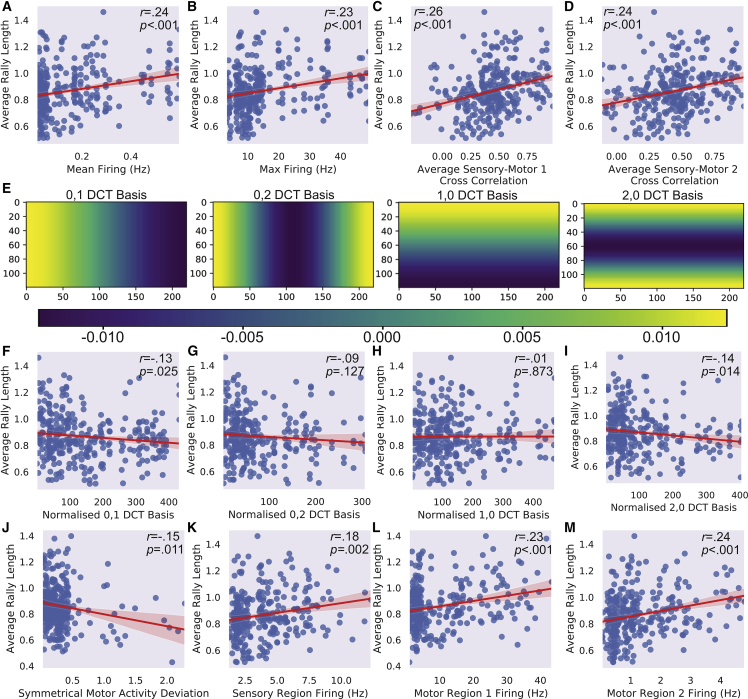
Relationship between electrophysiological activity and average rally length 302 gameplay sessions were analyzed after filtering outliers (*Z* score > ±3.29) from rallies with n = 30 biological replicates. (A) The mean spontaneous activity (Hz) over all electrodes showed a significant positive correlation with average rally length. (B–D) Similarly, the max spontaneous firing (Hz) also showed a significant positive correlation with average rally length. In line with this, the average cross correlation between the sensory region and both motor region 1 (C) and motor region 2 (D) had a significant positive correlation with average rally length. (E) The DCT scores of four different basis functions were calculated to quantify asymmetry in spontaneous activity. DCT scores were normalized to mean activity. The scale bar shows the value assigned to activity in the given area, where each DCT basis function quantifies a different type of asymmetry per pixel from −0.010 to 0.010. (F–H) Displays the significant negative correlation between DCT 0,1 and average rally length, showing that asymmetry on the horizontal axis is related to poorer performance. There was no significant relationship between DCT 0,2 (G), which measured asymmetry on the horizontal extremes compared with the center, or DCT 1,0 (H), which measured asymmetry on the vertical axis. (I–M) DCT 2,0 function displayed a significant negative correlation with average rally length, suggesting that asymmetry on the vertical edges compared with the middle was linked to poorer gameplay performance. In line with this, (J) displays the calculated deviation from symmetry in activity between motor regions during gameplay and finds a significant negative association, where greater asymmetry was linked to lower average rally lengths. Similarly, during gameplay the activity in the sensory (K), motor region 1 (L), and motor region 2 (M) all showed significant positive correlations with average rally length.

Exploratory uncorrected Pearson’s correlations were computed for key electrophysiological activity metrics and average rally length. A significant positive correlation was found between average rally length with mean (Figure 8A) and max (Figure 8B) firing. Likewise, the cross-correlations with the sensory region for both motor region 1 (Figures 8C) and 2 (Figure 8E) were significantly positively correlated with performance, further suggesting that robust connectivity is linked with better gameplay outcomes. To further investigate whether the topographical distribution of activity correlated with performance, the absolute values of four discrete cosine transform (DCT) coefficients normalized to mean activity were used to summarize spatial modes of spontaneous activity and assess the symmetry of activity (Figure 8E). DCT 0,1, which measures activity across the horizontal plane (Figure 8F), and DCT 2,0, which measures activity on the horizontal edge versus the horizontal center (Figure 8I), were significantly negatively correlated with average rally length. Yet, DCT 0,2, which shows difference between activity on the vertical edges and the vertical center (Figure 8G), and DCT 1,0 which measures activity across the vertical plane (Figure 8H), did not significantly correlate. Given configuration layout, it is coherent that gameplay performance is closely linked to deviations in symmetry of electrophysiological activity. To confirm the importance of symmetry, gameplay electrophysiological activity was analyzed for both motor regions, and the normalized deviation away from symmetry was calculated. As deviation away from symmetry resulted in a significant negative correlation with the average rally length, any asymmetry exceeding approximately 1 deviation appeared to completely prevent performance above that observed in controls (Figure 8J). This suggests a limit to which cultures can self-organize spontaneous activity if cell culture quality is uneven. Finally—in line with the results above—higher activity in the sensory region (Figure 8K), motor region 1 (Figure 8L), and motor region 2 (Figure 8M) during gameplay was also correlated with higher average rally lengths.

## Discussion

Here, we present the *DishBrain* system, a system capable of embodying BNNs from various sources in a virtual environment and measuring their responses to stimuli in real time. The ability of neurons, especially in assemblies, to respond to external stimuli adaptively is well established *in vivo* as it forms the basis for all animal learning (Attinger et al., 2017). However, this work is the first to establish this fundamental behavior *in vitro* for a goal-directed behavior. We were able to use this silico-biological system to investigate the fundamentals of biological neuronal computation. In brief, we introduce the first SBI device to demonstrate adaptive behavior in real time. The system itself offers opportunities to expand upon previous *in silico* models of neural behavior, such as where models of hippocampal and entorhinal cells were tested in solving spatial and non-spatial problems (Whittington et al., 2020). Minor variations on the *DishBrain* platform, selected cell types, drug administration, and feedback conditions would enable an *in vitro* test to garner data on how cells process and compute information that was previously unattainable.

Most significantly, this work presents a substantial technical advancement in creating closed-loop environments for BNNs (Bakkum et al., 2008a; Chao et al., 2008; Wagenaar et al., 2004). We have emphasized the requirement for embodiment in neural systems for goal-directed learning to occur. This is seen in the relative performance over experiments, where denser information and more diverse feedback impacted performance. Likewise, when no feedback was provided yet information on ball position was available, cultures showed significantly poorer performance and no learning. Of particular interest was the finding that when stimulatory feedback was removed and replaced with silent feedback (i.e., transient removal of all stimuli), cultures were still able to outperform those with no feedback as in the open-loop condition, albeit to a lesser extent. One interpretation is that playing “Pong” generates more predictable outcomes than not playing “Pong” by reducing uncertainty. Note that a “miss” results in unpredictable outcomes because the ball resets and its subsequent motion is unpredictable. In terms of the informational entropy of the stimulus being delivered, while an unpredictable stimulus would have high entropy, the silent condition still entails higher entropy relative to successful play as the ball restarts in a random direction. This is consistent with our results, as the more unpredictable an outcome, the greater the observed learning effect—as the BNN learns to avoid uncertainty.

It is interesting to note, however, that the internal information entropy of BNN activity does not exactly mirror the information entropy of the external stimulation: while the unpredictable stimulus increased internal entropy, so did the Silent condition feedback. However, for a BNN to alter activity in response to feedback, there must be a change to its sensory input observable by the system that can be associated with its previous activity. This is consistent with the absence of learning in the open-loop/No-feedback condition, which by its nature affords no opportunity for learning, and likewise showed higher internal information entropy than the other two feedback conditions. This supports the thesis that stimulation alone is insufficient to drive learning: there must be a motivation for learning behaviors that influence the (external) observable stimulus. When faced with unpredictable sensorium, playing “Pong” successfully acts as a free energy-minimizing solution. Even if the internal information entropy of a system is increased following feedback and has lower external information entropy (e.g., silent feedback), this may not provide the same impetus for learning. These findings accord with the proposed role of a Markov blanket, providing a statistical boundary of the system to separate it into internal and external states (Kirchhoff et al., 2018; Palacios et al., 2020). Yet simply minimizing entropy (i.e., average surprise) may offer an overly simplified account of adaptive behavior: a key aspect of active inference is the selection of actions that minimize the surprise or free energy expected on following that action. While these results are interesting and supportive, they are not conclusive, and future work is required, including exploring BNN behavior with a generative model.

Mechanistically, we sought to demonstrate the utility of the *DishBrain* by testing base principles that underwrite active sensing via the FEP. The closest previous work examined blind source separation in neural cultures, yet did so in an open-loop context without physiologically plausible training (Isomura et al., 2015; Isomura and Friston, 2018). We show that supplying unpredictable sensory input following an “undesirable” outcome and providing predictable input following a “desirable” one significantly shapes the behavior of neural cultures in real time. The predictable stimulation could also be read as a process of stabilizing synaptic weights in line with previous research as it has been shown that higher firing rates augment short- and long-term potentiation (Pariz et al., 2018; Zhu et al., 2015). In a complementary fashion, the unpredictable stimulation could be seen by destabilizing connectivity by destroying undesirable free energy minima. These results could be understood as part of a dynamic interaction between layers of interacting Hebbian and homeostatic plasticity that could lead to increasing the likelihood of activity following certain stimulation patterns (Ly et al., 2012; Pariz et al., 2018; Toyoizumi et al., 2014). This accords with the increased functional plasticity observed during gameplay versus during rest. This may be a potential mechanism behind the FEP account of biological self-organization, sometimes discussed in terms of self-organized instability termed “*autovitiation*” (Friston et al., 2012).

Active cortical cultures, from both human and mouse cell sources, displayed synchronous activity patterns in line with previous research (Kamioka et al., 1996; Sakaguchi et al., 2019; Shi et al., 2012; Wagenaar et al., 2006). Importantly, significant differences between cell sources were observed, with HCCs outperforming MCCs (with nuances), on average, in gameplay characteristics. Although further work is required as this finding was auxiliary to the aim of the study, this is the first work finding functional, albeit preliminary, empirical evidence supporting the hypothesis that human neurons have superior information-processing capacity over rodent neurons (Beaulieu-Laroche et al., 2018; Mihaljević et al., 2020). Previous work has proposed that biophysical structures in human cells compared with mouse cells would yield different input-output properties and may thereby explain different computational capacities (Poirazi and Papoutsi, 2020). When focusing on the initial development of the system, we could not feasibly and empirically test all key aspects, such as differences in cell sub-types, microscopic cell structure, or interneuron density. However, the opportunity exists for future studies to focus on elucidating these differences. The *DishBrain* system described in this work potentially offers the first avenue to accurately assess differences in neurocomputational ability, making this an exciting area of future research.

Another finding from this work relates to innate cell network organization, seen in the definition of motor regions. Our early pilot studies, along with previous work in this field (Bakkum et al., 2008a), mapped motor regions based on network activity scans. However, we were interested in the extent that self-organization would adapt if sensory and motor regions were fixed between cultures. Our findings demonstrate that while significant self-organization of activity can occur, this was limited when active cells were not evenly distributed across the MEA. The changes in activity during gameplay are consistent with past work showing that feedback between environment and action is required for proper *in vivo* neural development (Attinger et al., 2017). The observed changes also suggest that perhaps this development occurs based on properties inherent at the level of the cell. While these conclusions are tentative as the statistics of stimulations do differ between control experiments, the data does highlight future research directions. Further experimentation on the extent that the closed-loop environment is important for learning should include increasing the delay between reading neural activity and having it influence the environment or using stimulation decoupled from the environment. Nonetheless, the *DishBrain* system and future improvements of this technology do provide the opportunity to explore network dynamics to better understand this aspect of self-organization and include investigations into structural organization of BNNs.

Due to current hardware limitations, the sensory stimulation is much coarser compared with that for even simple *in vivo* organisms. This meant that it was not possible to distinguish, in real time, between stimulation of neuronal somatic or dendritic domains and that both were likely stimulated. Likewise, it was not computationally possible in real time to separate processing electrical changes from different neuronal structures such as discriminating between action potentials from the soma versus dendrites. Improving both areas is a key direction for future research. Additionally, it was infeasible to meaningfully implement mechanisms that would be crucial for an *in vivo* organism attempting a comparable task, such as proprioception, or to decouple the closed-loop system to test the impact of time delays. Moreover, the relatively small number of cells embedded in a monolayer format means the neural architecture driving this behavior is incredibly simple in terms of the number of possible connections available compared with even small organisms that have a 3D brain structure. Nonetheless, using only simple patterns of predictable and unpredictable stimulation, this system was able to show systematic behavior in an order of minutes. While within-session learning was well established, between-session learning over multiple days was not robustly observed. Cultures appeared to relearn associations with each new session. Given that cortical cells were selected, this is to be expected as *in vivo* cortical cells are not specialized for long-term memory (Rolls, 2018). Future work with this system can investigate the use of other neuronal cell types and/or more complex biological structures.

### Conclusion

Using this *DishBrain* system, we have demonstrated that a single layer of *in vitro* cortical neurons can self-organize activity to display intelligent and sentient behavior when embodied in a simulated game-world. We have shown that even without a substantial filtering of cellular activity, statistically robust differences over time and against multiple controls could be observed in the behavior of neuronal cultures in their sensed world. These findings provide a promising demonstration of an SBI system that learns over time in a systematic manner directed by input. The system provides the capability for a fully visualized model of learning, where unique environments may be developed to assess the actual computations being performed by BNNs. This is something that is long sought after and extends beyond purely *in silico* models or predictions of molecular pathways alone (Karr et al., 2012; Whittington et al., 2020; Yu et al., 2018). Therefore, this work provides empirical evidence that can be used to support or challenge theories explaining how the brain interacts with the world and intelligence in general (Friston, 2010; Schwartz, 2016). Ultimately, although substantial hardware, software, and wetware engineering are still required to improve the *DishBrain* system, this work does evince the computational power of living neurons to learn adaptively in active exchange with their sensorium. This represents the largest step to date of achieving SBI that responds with externally defined goal-directed behavior.

## STAR★Methods

### Key resources table

**Table:** 

REAGENT or RESOURCE	SOURCE	IDENTIFIER
**Antibodies**		

Synapsin1(1:500; Rabbit)	Abcam, Cambridge, MA, USA	RRID: AB_2920663
NeuN (1:500; Rabbit)	Abcam, Cambridge, MA, USA	RRID: AB_10711153
Beta-III Tubulin (1:500; Mouse)	Kenilworth, NJ, USA	RRID: AB_2210524
MAP2 (1:1000; Chicken)	Abcam, Cambridge, MA, USA	RRID: AB_2138153
TBR1 (1:200; Rabbit)	Abcam, Cambridge, MA, USA	RRID: ab183032: https://www.abcam.com/tbr1-antibody-epr81382-ab183032.html
GFAP (1:500; Chicken)	Abcam, Cambridge, MA, USA	RRID: AB_304558
KI67 (1:500; Mouse)	Abcam, Cambridge, MA, USA	RRID: ab245113: https://www.abcam.com/ki67-antibody-37c7-12-ab245113.html
DAPI(1:1000)	Abcam, Cambridge, MA, USA	RRID: ab228549: https://www.abcam.com/dapi-staining-solution-ab228549.html
Goat polyclonal Secondary Antibody to Chicken IgY (Alexa Fluor® 555; 1:500)	Abcam, Cambridge, MA, USA	RRID: AB_2893330
Goat Anti-Rabbit IgG H&L (Alexa Fluor® 488; 1:500)	Abcam, Cambridge, MA, USA	RRID: AB_2630356
Goat Anti-Mouse IGG (CF 647; 1:500)	Sigma-Aldrich PTY. LTD., NSW, Australia	RRID: SAB4600183: https://www.sigmaaldrich.com/AU/en/product/sigma/sab4600183

**Bacterial and virus strains**		

vSVG (Envelope)	Zhang et al. (2013)	Addgene #8454
RSV (CMV - Rev)	Zhang et al. (2013)	Addgene #12253
pMDL (CMV - Gag and Pol)	Zhang et al. (2013)	Addgene #12251
FUW-M2rtTA	Zhang et al. (2013)	Addgene #20342
FUW-TetO-Ngn2-P2A-puromycin	Zhang et al. (2013)	Addgene #52047

**Chemicals, peptides, and recombinant proteins**		

SB431542	Stemcell Technologies Australia, Melbourne, Australia	72232
LDN193189	Stemcell Technologies Australia, Melbourne, Australia	72147

**Deposited data**		

Raw data	This paper	https://gitlab.com/PaperReview/2021-07-12251

**Experimental models: Cell lines**		

control hiPSC line (ATCC® PCS-201-010)	Gene editing facility at the Murdoch Children’s Research Institute	ATCC® PCS-201-010
RM3.5 GT-GFP-01	Kao et al. (2016)	n/a

**Software and algorithms**		

ImageJ-FIJI	NIH	SCR_002285
BioRender	BioRender	SCR_018361
MaxLab Live Software	MaxWell Biosystems	https://doi.org/10.1039/C5LC00133A
Python	Python Software Foundation	RRID: SCR_008394; https://www.python.org/download/releases/3.0/
Custom Python Analysis	This paper	https://doi.org/10.17605/OSF.IO/5U6QV
Desposited data	This paper	https://doi.org/10.17605/osf.io/5u6qv

### Resource availability

#### Lead contact

Requests for further information and other correspondence should directed to and will be fulfilled by the lead contact, Dr Brett J. Kagan (Brett@CorticalLabs.com).

#### Materials availability

This study did not generate new unique reagents.

### Experimental model and subject details

#### Ethics statement

All experimental procedures were conducted in accordance with the Australian National Statement on Ethical Conduct in Human Research (2007) and the Australian Code for the Care and Use of Animals for scientific Purposes (2013). Animal work was conducted under ethical approval E/1876/2019/M from the Alfred Research Alliance Animal Ethics Committee B. Experiments were performed at Monash University, Alfred Hospital Prescient with the appropriate personal and project licences and approvals. Work done using hiPSCs was in keeping with the described material transfer agreement below.

#### Animal breeding and maintenance

BL6/C57 mice were mated at Monash Animal Research Platform (MARP). Upon confirmation of pregnancy, animals were transported via an approved carrier to the Alfred Medical Research and Education Precinct (AMREP). Pregnant animals were housed in individually ventilated cages until the date when they were humanely killed, and primary cells were harvested.

#### Stem cell lines

Initial work was conducted using a control hiPSC line supplied by the Gene Editing Facility at the Murdoch Children’s Research Institute (ATCC® PCS-201-010) from an ATCC PCS-201-010 background and transferred under a Material Transfer Agreement. ATCC line has been validated as per https://www.atcc.org/products/pcs-201-010 and comes from an XY donor isolated from neonatal foreskin. Later work involved an hiPSC lines used in this work constitutively expressing fluorescent reporters under control of the glyceraldehyde 3-phosphate dehydrogenase (GAPDH) promoter (cell lines were generated by Professor Edouard G. Stanley and colleagues from the Murdoch Children’s Research Institute and provided under a Material Transfer Agreement) (Kao et al., 2016). The GAPDH gene encodes a protein critical in the glycolytic pathway, whereby ATP is synthesised from glucose. As this function is highly conserved across multiple cell types GAPDH is ubiquitously expressed at high levels across multiple cell types, making it a suitable gene for which to base a gene-expression system (Barber et al., 2005). RM3.5 line validation is reported in (Barber et al., 2005) and comes from an XY donor isolated from neonatal foreskin. This transgene expression system, termed GAPTrap, involves the insertion of the specific reporter gene into the GAPDH locus in hiPSCs using gene-editing technology (Kao et al., 2016). For this study, RM3.5 GT-GFP-01 constitutively expressing green fluorescent protein under the GAPDH promoter was utilised. The RM3.5 hiPSC line was initially derived from human foreskin fibroblasts and reprogrammed using the hSTEMCCAloxP four factor lentiviral vector as reported previously (Somers et al., 2010). All procedures described below were applied to be both cell lines. Both lines were maintained in an undifferentiated, pluripotent state in a feeder-free system using E8 media (Thermo Fisher Scientific, Carlsbad, USA) supplemented by a Penicillin/streptomycin solution at 5 μL/mL. Cells were plated on T25 353108 Blue Vented Falcon Flasks (Corning, Durham, USA) that were coated approximately 1 h prior with extracellular matrix vitronectin (Thermo Fisher Scientific, Carlsbad, USA).

#### Stem cell growth and maintenance

All procedures were carried out using sterile techniques. Prior to passaging, cell confluence was recorded and the required split ratio was determined. Media was aspirated from cells and cells were washed with 5 mL of PBS −/− before passaging to remove detached cells and other debris. 3 mL of a 0.05 μM EDTA in PBS −/− was used for the dissociation and passaging of hiPSCs as aggregates without manual selection or scraping, was added to cells, and allowed to incubate at 37°C for approximately 3.5 min. After visual examination using 10X microscope indicated that cells had lost sufficient adhesion, EDTA was aspirated, and blunt trauma applied to base of the T25 flask to dislodge cells. Cells were suspended in 2 mL E8 and transferred to 15 mL falcon tube. As described above, vitronectin coated T25 flasks were prepared and aspirated before the addition of 5 mL of E8 solution. Approximately 1:10 of evenly distributed cell suspension was added to the prepared T25 flask. The flask was then gently swirled to ensure even distribution before being incubated overnight at 37°C. Media was changed daily.

### Method details

#### Primary cell culturing

Cortical cells were disassociated from the cortices of E15.5 mouse embryos. Embryos were decapitated, and with a stereotactic microscope, the skin, bone and meninges were removed, and the anterior cortex dissected out. Approximately 800,000 cells were plated down onto each pre-prepared HD-MEA. Cultures began to upregulate spontaneous activity and display synchronised firing around DIV 10 at which point they were used for experimentation.

#### Stem cell dual SMAD differentiation

Cellular differentiation followed a titrated dual SMAD inhibition protocol for the generation of cortical cells from pluripotent cells established by the Livesey group with minor adjustments as represented in Figures S5B (Shi et al., 2012). Cells were plated in 24 well plates coated with human laminin H521. When cells reached ≈80% confluency, neural induction was initiated by using standard neural maintenance (N2B27) Base Media with 100 ng/mL LDN193189 (Stemcell Technologies Australia, Melbourne, Australia) and 10 μm SB431542 (Stemcell Technologies Australia, Melbourne, Australia). Media was changed every day from day 0 to day 12. After appearance of neural rosettes and initial passaging standard N2B27 media with FGF2 20 ng/ml was utilised from day 12 to day 17 to achieve a dorsal forebrain patterning. Cells were then expanded and deemed ready for plating onto MEA or slides based on morphology at approximately 30–33 days. On the day of transplant, cells were detached with Accutase (Stemcell Technologies Australia, Melbourne, Australia) to a single cell suspension and centrifuged at 300*g*. The cell pellet was resuspended at 10,000 cells/μl in BrainPhys (Stemcell Technologies Australia, Melbourne, Australia) neural maintenance media with Rho Kinase Inhibitor IV (Stemcell Technologies Australia, Melbourne, Australia; 1:50 dilution) with approximately 10^6^ cells plated onto each MEA. Cells began to display early but widespread spontaneous activity around DIV 80, at which point they were ready for experimentation.

#### Stem cell NGN2 direct differentiation

Cortical excitatory neurons were generated by the expression of NGN2 in iPSCs. iPSCs were plated at 25,000 cells/cm^2^ in a 24-well plate coated with 15 μg/ml human laminin (Sigma, USA). The following day, cells were transduced with NGN2 lentivirus (containing a tetracycline-controlled promoter coupled with a puromycin selection cassette) in combination with a lentivirus for the rtTA (reverse tetracycline-controlled transactivator). NGN2 gene expression was activated by the addition of 1 μg/ml doxycycline (Sigma, Australia), this was referred to as differentiation day 0. Cells were cultured in neural media consisting of 1:1 ratio of DMEM/F12:Neurobasal media supplemented with (all reagents from Thermofisher, USA) B27 (#17504-044), N2 (17,502-048), Glutamax (#35050-060), NEAA (#11140-050), β-mercaptoethanol, ITS-A (#51300-044) and penicillin/streptomycin (#15140-122). On Day 1, 1.0 μg/mL puromycin (Sigma, Australia) was added for 3 days at which point neurons were supplemented with 10 μg/ml BDNF (Peprotech, USA) and lifted with accutase, in preparation for plating on HD-MEA chips. HD-MEA chips were pre-treated with 100 μg/ml PDL (Sigma, USA) and 15 μg/mL laminin (Sigma, USA). For each well 1x10^5^ NGN2 induced neurons at DD4 were combined with 2.5x10^4^ primary human astrocytes (ScienceCell, USA) in each well of the MEA plate. To arrest cell division of astrocytes 2.5 μM Ara-C hydrochloride (Sigma, USA) was added at day 5 for 48 h. Cells were maintained in neural media supplemented with BDNF and media changed at least 1 day prior to recordings.

#### HEK293T cell culturing

Human Embryonic Kidney Cells 293T (HEK 293T; Merck KGaA, Darmstadt, Germany), were cultured in DMEM (Thermofisher Scientific, USA) supplemented with 10% fetal bovine serum (Thermofisher Scientific, USA) under standard conditions. Cells were used as a non-neural control and plated onto MEA as described below with the exception that testing began 24 h after plating as this cell type does not mature into electrically active cells.

#### MEA setup and preparation

MaxOne Multielectrode Arrays (MEA; Maxwell Biosystems, AG, Switzerland) were used for this research. The MaxOne is a high-resolution electrophysiology platform featuring 26,000 platinum electrodes arranged over an 8 mm^2^. The MaxOne system is based on complementary meta-oxide-semiconductor (CMOS) technology and allows recording from up to 1024 channels. Stimulation was theoretically possible up to 32 electrodes. In practice it was not possible to route 32 electrodes through independent stimulation units to facilitate independent electrode level control, especially if these electrodes were spatially proximate to each other. This meant that for the actual setup of input stimulation described below a subset would be limited by the desired spatial configuration – in this case to 8 individually controlled electrodes. MEAs and chambered glass slides are coated with either polyethyleneimine (PEI) in borate buffer for primary culture cells or Poly-D-Lysine for cells from an iPSC background before being coated with either 10 μg/ml mouse laminin or 10 μg/ml human 521 Laminin (Stemcell Technologies Australia, Melbourne, Australia) respectively to facilitate cell adhesion.

#### Plating and maintaining cells on MEA

Approximately 10^6^ cells were plated on MEA after preparation via method already described. Cells were allowed approximately one hour to adhere to MEA surface before the well was flooded. The day after plating, cell culture media was changed to BrainPhys™ Neuronal Medium (Stemcell Technologies Australia, Melbourne, Australia) supplemented with 1% penicillin-streptomycin. Cultures were maintained in a low O_2_ incubator kept at 5% CO_2_, 5% O_2_, 36°C and 80% relative humidity. Every two days, half the media from each well was removed and replaced with free media. Media changes always occurred after all recording sessions.

#### Measuring of electrophysiological activity

Licensed MaxLab Live Scope V20.1 software was used to run activity scans. Checkerboard assays consisting of 14 configurations at 15 seconds of spike only record time were run daily immediately preceding the running of the DishBrain software. Gain was set to 512x with a 300 Hz high pass filter. Spike threshold was set to be a signal six sigma greater than background noise as per recommended software settings. Mean, max and variance of both amplitudes and firing rates was extracted from these assays and mapped using custom software: the first nine components of discrete cosine transform basis functions of space were used to summarise the spatial profile of spiking activity. The ensuing coefficients were then used in subsequent correlation analyses.

#### *DishBrain* software platform

The current *DishBrain* platform is configured as a low-latency, real-time MEA control system with on-line spike detection and recording software, see Figure S3. The *DishBrain* software is controlled by a low latency, real-time piece of software named ‘*DishServer’*, which replaces and extends a corresponding piece of MaxWell vendor software called ‘*MXWServer’*. *DishServer* is capable of receiving voltage readings from MaxOne vendor hardware, processing these readings, simulating a virtual environment, encoding the results as MaxOne electrode commands, and sending these commands back to the MaxOne hardware. When run on a computer with access to a MaxOne hardware setup with a live culture in place, the system acts as a closed loop that we can configure and record for analysis. Working closely with MaxWell Biosystems we enabled capabilities not available using the native vendor software. The MaxOne MEA is configured to read up to a particular 1024 of its 26,400 electrodes, at a rate of 20,000 samples per second. As shown in Figure S2B, these samples are optionally recorded as-is, for later analysis, but are also run through a sequence of computationally efficient Infinite Impulse Response (IIR) filters to calculate noise and activity levels, which are compared in order to detect spikes. Incoming samples are filtered with a 2nd order high-pass Bessel filter with 100Hz cut-off, the absolute value is then smoothed using a 1st order low-pass Bessel filter with 1Hz cut-off, the spike threshold is proportional to this smoothed absolute value.

#### Representation of the gameplay environment

Spikes are themselves optionally recorded in binary files, and regardless of recording are counted over a period of 10 milliseconds (200 samples), at which point the game environment is given the number of spikes detected in each of the configured electrodes in predefined motor regions as described below. These spike counts are interpreted as motor activity depending on which motor region the spikes occurred in, thereby moving the ‘paddle’ up or down in the virtual space. At each of these 10ms intervals the pong game is also updated, with a ball moving around a play area at a fixed speed, ‘bouncing’ off the edges of the play area and off the paddle, until it hits the edge of the play area behind the ‘paddle’, which marks the end of one ‘rally’ of pong. At the end of the rally, the game environment will instead configure the stimulation sequencer to apply one of three types of feedback described below: random, silent or none. Under the standard stimulus condition, feedback is also provided when the ball contacts the paddle as described below. As described in detail below, during each rally the location of the ball relative to the paddle is encoded as stimulation to one of eight stimulation sites, which is tracked in an internal ‘stimulation sequencer’ module. The stimulation sequencer is updated 20,000 times a second, once every time a sample is received from the MEA, and once the previous lot of MEA commands should have finished, it constructs another sequence of MEA commands based on the place-code and rate-code information that it has been configured to transmit. The stimulations take the form of a short square bi-phasic pulse that is a positive voltage, then a negative voltage. A Digital to Analog Converter (or DAC) on the MEA will read and apply this pulse sequence to the given electrode. Figure S5C shows an image of the game visualiser, and a real-time interactive version is available Video S2 at https://spikestream.corticallabs.com/. There was also the option to record cells at ‘rest’ where a gameplay environment was initiated and activity was recorded to move the paddle, but no stimulation was delivered, with corresponding outcomes still being recorded. This acted as a baseline control to determine the gameplay characteristics of a culture based on spontaneous activity alone.

#### Interface with Maxwell API

To interface with Maxwell API, *DishBrain* uses a negative DAC value first because this corresponds to a positive voltage in the MaxWell API. Finally, the spike detection is also capable of ‘blinding’, which is expected to occur after each stimulation; in order to prevent DAC stimulation from being interpreted as neuron activity, all 1024 channels are ignored for a configurable number of samples, after either detecting anomalous activity directly, or after receiving acknowledgement from the MEA that a DAC command has been executed. The existing API was used only for loading configurations. Low level code was written in C to allow for minimal processing latencies—so that packet processing latency was typically <50 μs. High level code, including configuration set ups and broader instructions for game settings were implemented in Python. This allowed a spike-to-stim latency of approximately 5ms, with the substantive delay due to inflexible hardware buffering built into MaxOne hardware.

#### Initial pilot testing

Initial tests were conducted to assay which input configurations cell cultures would survive. Testing time was found to be a highly sensitive parameter, as cells did not tolerate testing times >1.5 h. When measurements were taken it was concluded that this was likely due to increased temperature in the cultured wells in which cells were plated in due to activity and the resulting increased evaporation and changes in osmolarity. Cells survived testing administration of stimulation up to 3000 mV for up to one hour which was the maximum testing time considered given the above findings. While this did create excess noise in recording cellular activity across the MEA during the stimulation period, there were no significant changes to spontaneous activity in the cell cultures before and after the period of stimuli administration. Initial experiments delivered purely place-coded stimulation, where the distance from the centre of the sensory area was interpreted as distance from the centre of the paddle aligning with the ball.

#### Pilot test with EXP3 algorithm

After initial pilot testing of the *DishBrain* system, two pathways were identified to modify performance: encoding of information and decoding of activity. For the latter, an Exponential-weight algorithm for Exploration and Exploitation (EXP3) algorithm was used during pilot study 2 only for the adaptive selection of electrode layouts, with the objective of optimising gameplay performance and determining whether key motor region definitions were on average more suitable for gameplay than others (Yang et al., 2020). These different configurations options are illustrated in Figure S3 (Seldin et al., 2012).This algorithm was implemented to maintain a list of weights for each action and was designed to minimise regret (the difference between the accumulated loss and the loss achieved) by preferencing electrode configurations which were associated with a higher probability of the ball being returned. EXP3 is robust to changes in the underlying distribution of returns; this is important because neurons are also concurrently learning, and their behavior changing over time. Optimising all possible assignments of electrodes to actions would require a prohibitively large set of choices, so a representative set of balanced layouts were used. EXP3 is an online optimisation algorithm for the "multi-armed bandit" problem. It selects between several discrete choices, over a series of rounds. Each discrete choice yields an observable stochastic loss. The best choice is never revealed, even post-hoc. Quality of choices can only be inferred from noisy returns - exploration and exploitation must be balanced. In this work, one of the discrete sets of electrode-action mappings called 'motor layouts' was chosen on each round. The loss to be minimized is calculated using the following equation:

(Equation 1) $$L_{i}=\frac{\min\left({\text{score}}_{i},10\right)}{10}-1$$

Where L_i_ is the loss at the end of the rally i and score_i_ is number of bounces during that rally. During the i-th rally, a given layout is used and is fixed during the entire rally. At the end of the rally, a different layout is chosen by EXP3 for the next rally and the game play continues. When using EXP3 the system can adaptively optimize performance by choosing from a fixed set of alternative motor layouts (Figure S3). At the same time, a new blinding method (consensus blind) based on blinding all signals when >15 simultaneous large (>75 mV) spikes were detected, was implemented to block stimulation delivered by the system from being registered as cellular activity. It was hypothesised that a lack of blinding administered signals may contribute to the apparent performance observed in controls in our pilot study. As described in the main text, Figure S5D and shown in Table S4, experimental chips with configurations that would enable lateral inhibition were found to be selected significantly more compared to other configurations resulting in an equal distribution (*χ2* = 35690.93, p < 0.0001), including those that were more simplified like that used in the pilot where activity on the left moved the paddle left and conversely for the right (Figure S3**: Configuration 0**) and would be most easily influenced by various sources of bias (Espinoza et al., 2018; Fan et al., 2020; Obermayer et al., 2018). When the frequency tables of these two distributions were compared, they were also found to be significantly different, (*χ2* = 15229.323, p < 0.0001). Considering these differences, for this specific pilot study it was not valid to compare experimental and control groups as they are operating off different types of configurations. Given the apparent preference for configurations that would allow processes such as lateral inhibition to occur in experimental chips, coupled with the concern of having different groups operating from different configurations, it was decided to select configuration 3 for all cultures going forward, as it was chosen most frequently by the EXP3 algorithm. Moreover, if consensus blinding behaved as expected, control chips should also show no preference. This led us to suspect that consensus blinding was ineffective and on further investigation, particularly when using a higher and variable frequency of sensory stimulation, we discovered more evidence of consensus blinding failing than our previous testing revealed. To counter this, a new blinding method was implemented, which was termed ‘command count blinding’. This method blinded our readout of all motor activity when a command was sent to generate any form of stimulation. During testing this was found to be significantly more robust than the previously used consensus blinding and allowed us to proceed with increasing the density and variability of sensory stimulation.

#### Input configuration

Stimulation is delivered at a given Hz and voltage as appropriate for the required input type across 8 predefined electrodes in a sensory area, as shown in Figure 4B. A total of 5 types of input were able to be delivered. This consisted of either “Sensory Stimulus” that encoded ‘ball’ position, or one of four feedback protocols, either Unpredictable, Predictable, Silent, or No-feedback.

##### Sensory stimulus

Given that cells appeared robust to voltage stimulation, the decision was made to base voltage levels on existing evidence of neurological function. Therefore, to prevent forcing hyperpolarised cells from firing, 75 mV was chosen as the sensory stimulation voltage that would relate to where the ball was relative to the paddle as described in the main text to key electrodes. For the main study, place coding was combined with a rate coding that delivered stimuli at 4 Hz when the ball was closest to the opposing wall and increased in a linear fashion to a max of 40 Hz as the ball reached the paddle wall.

##### Unpredictable stimulus

For the standard stimulus feedback condition unpredictable stimulation was delivered to the cultures when a ‘miss’ occurred – i.e., when the culture failed to line the ‘paddle’ up to connect with the ‘ball’. In order to add unpredictable external stimulus into the system, this feedback stimulus was set at 150 mV voltage and 5 Hz. This stimulation occurred at random sites at a random timescale over the 8 predefined input electrodes, for a period of four seconds, followed by a configurable rest period of four seconds where stimulation is paused, followed then by the next rally. Theoretically the higher voltage than that used for the Sensory Stimulus would be sufficient to force action potentials in cells subjected to the stimulation regardless of the state the cell was in, thereby being even more disruptive to the culture.

##### Predictable stimulus

For the standard stimulus feedback condition a predictable stimulation was delivered to cultures when a ‘hit’ occurred – i.e., when the cultures successfully lined up the ‘paddle’ to connect with the ‘ball. This was delivered at 75mV at 100Hz over 100ms. This occurred at the instant of when the simulated ball impacted the paddle and replaced other sensory information for the 100ms period. Predictable stimulation occurred at this frequency and period across all 8 stimulation electrodes simultaneously.

##### Silent feedback

Silent feedback only occurred for follow up studies in the Silent condition. This feedback replaced the Unpredictable Stimulus described above with no stimulation for the same length of time. Predictable Stimulus feedback was also removed during Silent Feedback sessions. This feedback is still distinct from No-Feedback as described below as it is a change in the culture environment that is tied to culture activity in a closed-loop manner and therefore a form of feedback.

##### No feedback

This condition only occurred for follow up studies in the No-feedback condition. This condition was designed to assess whether sensory stimulation was sufficient to drive learning in cultures and was an open-loop condition. This means that no feedback of any kind was delivered to the cultures based on any outcome or action. Standard Sensory Stimulus as described above was delivered to the cultures and the outcome was measured on the same metric, however when a ‘miss’ would normally occur, instead the ball continued the same trajectory bouncing off the wall behind the paddle – still recorded as a ‘miss’ – that would otherwise result in the end of a rally. When the ‘ball’ connected with the simulated paddle a ‘hit’ would be recorded. As such, under No-Feedback the entire gameplay session is essentially a single rally with the final position of the simulated ball being predictable from the initial vector, but with the scoring occurring as normal otherwise.

#### Output configuration

A total of 1024 electrodes were routed on the HD-MEA to record activity in a pattern as shown in Figure 4B. The ‘Sensory’ area, where stimulation electrodes were embedded as described above consisted of 626 electrodes. The remaining output electrodes were divided into predefined motor regions on the MEA, consisting of four regions that were defined either as motor region 1 or motor region 2 as shown in Figure 4B. As described above, this configuration was selected as it offered the possibility for biologically relevant features and minimized the chance of apparently successful performance through bias alone—as it precludes a direct relationship between input stimulation and output activity recording. Only activity in motor regions contributed towards paddle movement. Activity in motor region 1 moved the paddle ‘up’ and activity in motor region 2 moved the paddle ‘down’. Activity was measured over these two regions, where the region with higher activity would move the paddle in a corresponding direction. This was found to be extremely sensitive to culture characteristics, where asymmetrical spontaneous spiking activity in cultures would cause the paddle to move swiftly in only one direction. However, due to the technical difficulty of culturing neurons with precisely balanced activity in both these regions it was found to be necessary to add ‘gain’ into the system. This gain function measured activity in both regions and added a multiplier to a target of 20 Hz. Activity >20 Hz was weighted by a correction factor >1, while activity <20 Hz was weighted by a correction factor <1. This would allow changes in activity in each given region to influence the paddle position, even if they displayed different latent spontaneous activity. No other filtering or machine learning style weights were applied to decode motor region activity, meaning there was no need for regularization or risk of over fitting as all learning was required to occur within the biological neural cultures.

### Quantification and statistical analysis

#### Sample size and blinding protocols

No statistical methods were used to predetermine sample size. As all work was conducted within controlled environments uninfluenced by experimenter bias, experiments were not randomized, and investigators were not blinded to experimental condition. However, conditions were blinded where possible before final analysis to limit bias during analysis. Figure S5A presents a schematic of the overall experimental setup.

#### Immunocytochemistry

Cells were washed three times with sterile PBS and then fixed using 4% PFA for 20 min. After washing, cells were blocked 0.3% Triton-X and 1% goat serum in PBS for 1 h. Primary antibodies specific for Synapsin1 (1:500; ab254349; Rabbit; Abcam, Cambridge, MA, USA), NeuN (1:500; ab104225; Rabbit; Abcam, Cambridge, MA, USA), Beta-III Tubulin (1:500; MAB1637, Mouse; Kenilworth, NJ, USA), MAP2 (1:1000; Chicken; ab5392; Abcam, Cambridge, MA, USA), TBR1 (1:200; ab183032; Rabbit; Abcam, Cambridge, MA, USA), GFAP (1:500; ab4674; Chicken; Abcam, Cambridge, MA, USA), and Ki67 (1:500; ab245113; Mouse; Abcam, Cambridge, MA, USA) were incubated overnight. After washing, secondary antibodies (chicken 555, rabbit 488, mouse 647; Abcam, Cambridge, MA, USA) were incubated for 2 h. This was followed by 10 min of DAPI Staining Solution in PBS (1:1000, ab228549, Abcam, Cambridge, MA, USA) after which point slides were cover-slipped with ProLong Gold Antifade Mountant (Thermo Fisher Scientific, Waltham, MA, USA) mounting media and allowed to dry for 48 h.

#### Scanning electron microscopy

At various designated endpoints, media was aspirated from the MEA wells and cells were fixed with 2.5% glutaraldehyde (Electron Microscopy Sciences, PA, USA) and 2% paraformaldehyde (Electron Microscopy Sciences, PA, USA) in a 1 M sodium cacodylate buffer for 1 h. They were then washed three times in 1M sodium cacodylate buffer before being post-fixed with 1% OsO_4_ in a 1M sodium cacodylate buffer for 1 h. OsO_4_ was removed and the fixed cells were washed with three times in milliQ water and dehydrated via an ethanol gradient exchange (30%, 50%, 70%, 90%, 100%, 100% v/v) for 15 min each. After dehydration, the cells were dried by hexamethyldisilazane (Sigma Aldrich, St. Louis, MO, USA) exchange (3 × 10 min), and then allowed to evaporate for 5–10 min. MEA chips were then affixed to an aluminium stub with carbon tape and sputter coated with 30 nm layer of gold using a BAL-TEC SCD-005 gold sputter coated. All procedures were performed at room temperature. Coated MEA chips were then imaged using a FEI Nova NanoSEM 450 FEGSEM operating with an acceleration voltage of 10 kV and a working distance of 12 mm. Images were analysed using ImageJ v.1.52k and false coloured using Adobe Photoshop.

#### Widefield fluorescence microscopy

Images were captured using a Nikon Ti-E upright light microscope equipped with a motorised stage. All widefield images were captured using a 20X objective.

#### Data analysis

Data was analysed using custom code written in Python. Error bars are described in captions, except where graphs are box and whisker plots, where the line is the median, box indicates lower quartile to upper quartile and error bars show the rest of the distribution excluding outliers. The illustrative data provided in the text and figures include means and standard deviations. An alpha of p < 0.05 was adopted to establish statistical significance, providing a 5% chance of a false positive error. Where suitable assumptions were met, inferential frequentist statistics were used to determine whether statistically significant differences existed between groups. All tests were two tailed tests for statistical significance. For related samples, *t*-tests or independent *T*-tests alpha values for significance were corrected via the Bonferroni method. For one-way analysis of variance (ANOVA) and the multivariate 2 x 3 repeated measures ANOVA, when a significant interaction or main effect was found, this was followed up with pairwise Games-Howell post hoc tests with Tukey correction for multiple comparisons. This was adopted as there were always differences between sample sizes and variance due to inclusion of in-silico controls. When examining spiking activity, for all stimulus condition gameplay activity the first 10 s was excluded as the system generated substantial noise while initialising. Four chips were not analysed as the data recording was initially not implemented for the very first series of experiments, the remainder were all included without exclusion. 100ms time-lagged cross-correlations were calculated between activity detected in the sensory region against activity detected in each motor region separately. This method has previously been established as a method to define functional connectivity (Mohseni Ahooyi et al., 2018). Both linear and nonlinear cross-correlations methods were explored and selected based on whether assumptions were met. However, it should be noted that comparable trends were observed with both methods. Given the large sample sizes, the linear rainbow test for linearity was predominately relied upon to determine significant variations away from linearity. The relationship between the sensory region did not show a significant degree of nonlinearity for both Motor Region 1 (p = 0.699; Figure S5E) and Motor Region 2 (p = 0.122; Figure S5F. As such Pearson’s linear correlation were used to quantify these relationships. When the binned correlations between the two motor regions were assessed (without a time lag to determine synchronised activity) it was found to show a significant degree of nonlinearity (p = 1.32^−50^; Figure S5G). For this relationship Spearman’s correlation was used. To quantify the changing relationship between time in minutes and the correlation between motor regions, linear regression was used with minutes as the predictive variable and the correlation as the dependent variable. Activity in each motor region was grouped into 1000ms bins and the number of exclusive events, where activity was detected in either Motor Region 1 or Motor Region 2 but not both, above noise (amplitude < -5μv) was calculated for both rest and gameplay conditions. This was then compared between cultures under the rest condition and during the gameplay condition. As seen in Figure 8E, four DCT basis functions were used to summarise spatial modes of spontaneous activity. Uncorrected pairwise Pearson’s correlations were used to test the relationship between the ensuing scores—along with max and mean firing rates (Hz) and electrophysiological activity during gameplay described above —with average rally length.

#### Calculation of information entropy

The spatial locations of recording electrodes were used to regionalize the entire MEA into 18 rectangular clusters of 50 neighbouring electrodes as shown in Figure S5. In every cluster, the spike time information from each of these electrodes were used to calculate the local binary entropy of the group of electrodes in time windows of 100ms. The binary entropy function, denoted *H*_*b*_(*p*), is defined as the entropy of a Bernoulli process with probability *p* of one of two values. Given *Pr*(*X* = 1) = *p*, then *Pr*(*X* = 0) = 1−*p*; with *X* = 1 indicating the presence of a spike in the current time bin. The entropy of *X* (in shannons) is given by:

$$Hb\left(p\right)=-p\cdot {\log\;}_{2}\left(p\right)-\left(1-p\right)\cdot {\log\;}_{2}\left(1-p\right)$$

where 0.log_2_0 is taken to be 0. Hence, we calculated the local entropy of each cluster of channels over time windows of 100ms. The mean value of the calculated entropies over time and over all the spatial clusters was then compared between sessions of active Gameplay with different feedback types and the Rest session recordings. The comparisons were also carried out for the mean entropy in separated groups of motor and sensory electrode clusters during Rest and Gameplay sessions.

#### Calculation of functional plasticity

Including spatial information for quantifying network plasticity has proven more reliable than simply utilizing firing rates as described (Chao et al., 2007). We adapted this method to compare training-induced plasticity with the baseline plasticity measured before training during Rest sessions, we used the centre of activity (CA), a related population coding, explicitly including electrode locations as a relevant variable (Bakkum et al., 2008b; Chao et al., 2007).

$$C_{A}=\left[C_{AX},C_{AY}\right]=\frac{\sum_{k=1}^{N}F_{k}\cdot \left[X_{k}-R_{X},Y_{k}-R_{Y}\right]}{\sum_{k=1}^{N}F_{k}}$$

The centre of activity (CA) is defined as the vector summation of the number of action potentials recorded on each electrode *k* (i.e., *Fk*) weighted by the spatial location of the electrode. [*Xk*, *Yk*] represent the coordinates of electrode *k* and the reference point coordinates, [*RX*, *RY*], were set as the bottom left corner of the MEA. *N* is the total number of electrodes recorded on the MEA. In order to investigate the presence of training-induced plasticity, the mean Euclidean distance of calculated CAs in consecutive 5 min time intervals during the Gameplay sessions to the centroid of CAs in all the recorded 10 min reference periods or Rest state spontaneous activity sessions before training was measured $\left(i.e.\;{\bar{C}}_{A_{\text{Gamplay }(\text{t,t}+5)}}-{\bar{C}}_{A_{\text{Rest }(\text{0,}10)}}\;;\;\forall t\in \left\{0,5\right\}\right)$. This was then compared to the mean Euclidean distance of CAs in 5 min intervals of Rest recordings to their own centroid which is again the mean of CAs during all the 10 min Rest session recordings from each culture on each experimental day (i.e.C¯ARest(t,t+5)−C¯C¯ARest(0,10);∀t∈{0,5}). This was then repeated for every culture on each experimental day. These measurements were used to quantify the change in CAs from a *pre-training* period to different *post-training* periods. The average of this distance from the Rest period centroid in the Gameplay sessions and Rest sessions were calculated. One-way ANOVA test was performed to determine the statistical significance of the differences between the two groups.

### Additional resources

A visualiser of the system in real-time is available at https://spikestream.corticallabs.com/.

## Data Availability

Data All data, including electrophysiological spike recordings and the raw data of gameplay metrics from virtual environment, have been deposited at Open Science Framework (OSF) and are publicly available. DOI is listed in the key resources table. Code All original Python and Matlab analysis code used to process and analyse deposited data have been deposited at Open Science Framework (OSF) and is publicly available. DOI is listed in the key resources table. Any additional information required to reanalyse the data reported in this paper is available from the lead contact upon request.

## Supplemental information

Document S1. Figures S1–S6 and Tables S1–S4

Document S2. Article plus supplemental information
