# Supplementary material for: In vitro neurons learn and exhibit sentience when embodied in a simulated game-world
Source: Neuron. 2022 Dec 7;110(23):3952–3969.e8. doi: 10.1016/j.neuron.2022.09.001 (PMC9747182; doi:10.1016/j.neuron.2022.09.001)
Supplement: Document S1. Figures S1–S6 and Tables S1–S4 [file mmc1.pdf]

Neuron, Volume *110*

Supplemental information

In vitro neurons learn and exhibit sentience when embodied in a simulated game-world

Brett J. Kagan, Andy C. Kitchen, Nhi T. Tran, Forough Habibollahi, Moein Khajehnejad, Bradyn J. Parker, Anjali Bhat, Ben Rollo, Adeel Razi, and Karl J. Friston

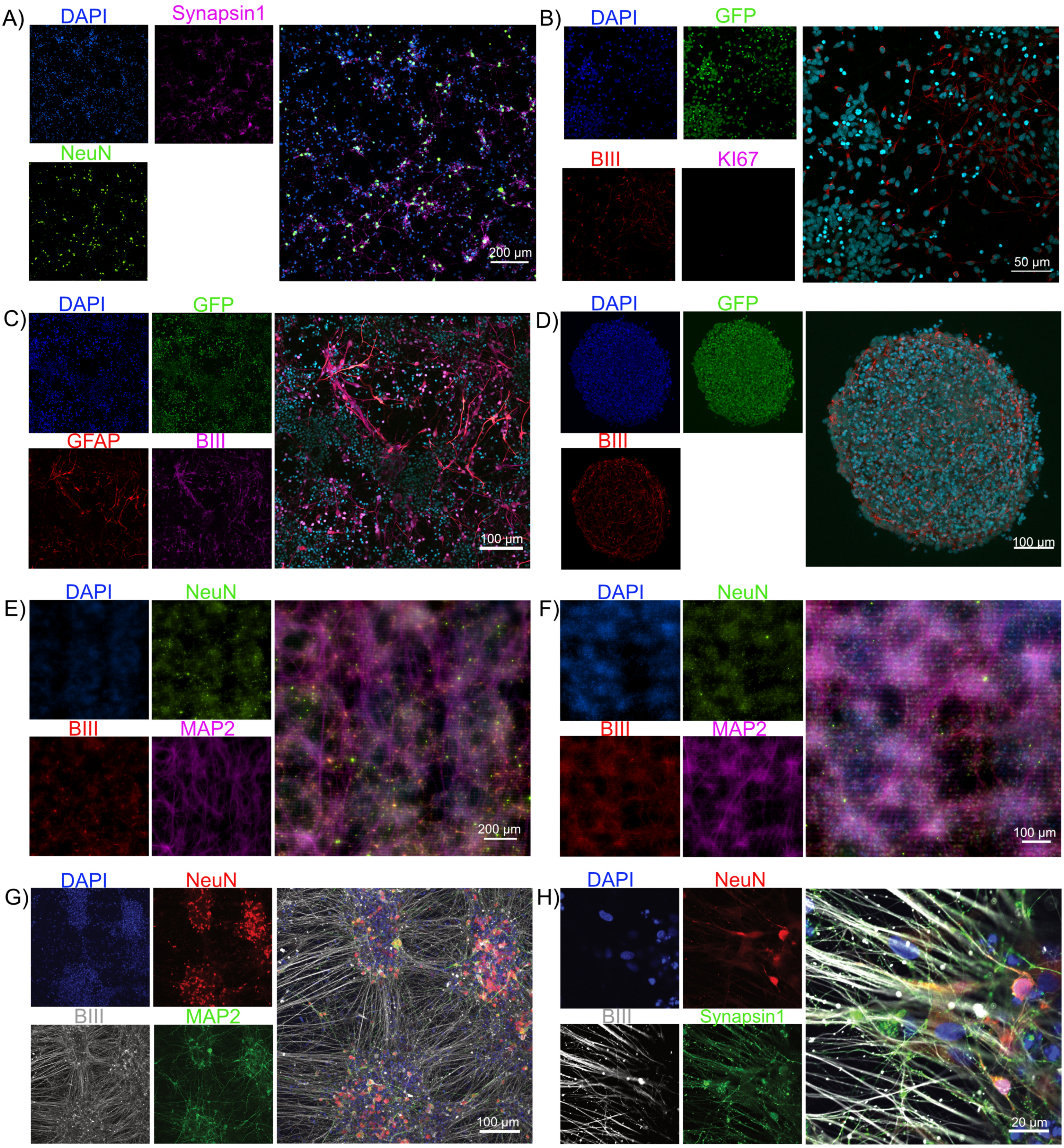

Fig. S1. Cortical neurons can be obtained via multiple methods, related to Fig. 2. Scale bars as shown on figure. **A)** Primary mouse cortical neurons show diverse expression of synapsin1 which marks synaptic vesicles and actin filaments across long reaching neural networks. **B)** – **F)** Shows that using a RM3.5 cell line comparable cortical cultures can be generated using the dual SMAD inhibition protocol described in Methods. **B)** Shows endogenous expression of GFP, beta3-tubulin (BIII) marking axons and a lack of Ki67 suggesting no dividing cells, **C)** additional shows these cells expressing GFAP for supporting glial cells Further images in **D)** show a characteristic neurosphere structure neurons would often spontaneously form when plated at high density, a dense pseudo three-dimensional sphere with dense connections of neurons and axons throughout. **E)** & **F)** display hIPCSs differentiated to neurons using the NGN2 method and mouse primary cortical neurons respectively, both plated of HD-MEA and allowed to mature before staining. These cells display all markers previously described, but due to the reflective material of the CMOS chip, it is infeasible to get high resolution fluorescent images of cells on the chips, leading to the adoption of SEM imaging shown in the main text. **G)** & **H)** also show hIPCSs differentiated to neurons using the NGN2 method; **G)** Staining of mature neural monolayer cultures with the majority of cells expressing NeuN which marks neuronal cells, MAP2 marks dendrites and β3-Tubulin which marks long-range axons. **H)** Further staining shows that along with β3-Tubulin these cells express the pre-synaptic marker synapsin1 across the soma and cell projections.

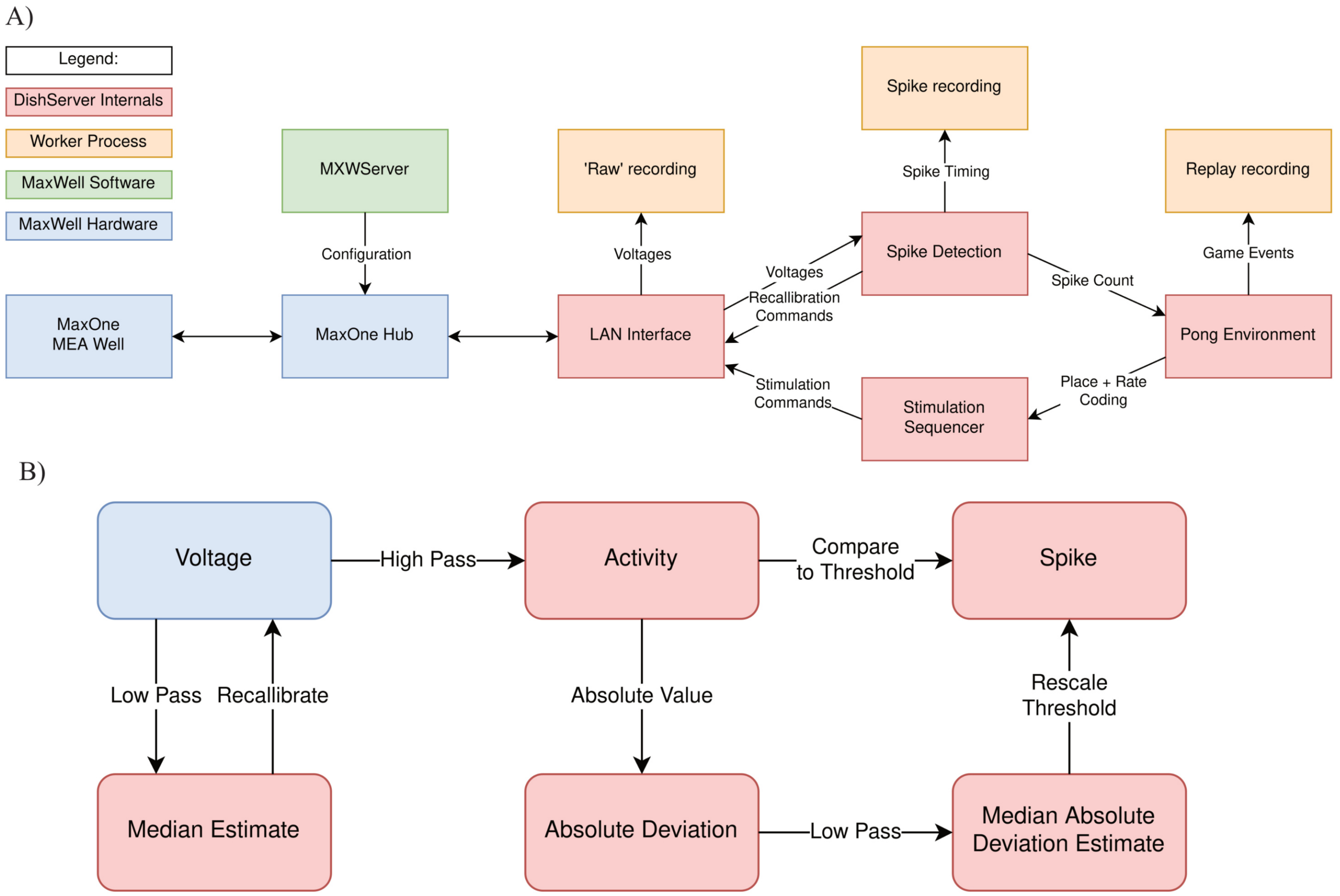

Fig. S2. Schematics of software used for *DishBrain,* related to Fig. 4B. A) Software components and data flow in the DishBrain closed loop system. Voltage samples flow from the MEA to the 'pong' environment, and sensory information flows from the 'pong' environment back to the MEA, forming a closed loop. The blue rectangles mark proprietary pieces of hardware from MaxWell, including the MEA well which may contain a live culture of neurons. The green MXWServer is a piece of software provided by MaxWell which is used to configure the MEA and Hub, using a private API directly over the network. The red rectangles mark components of the 'DishServer' program, a high-performance program consisting of four components designed to run asynchronously, despite being run on a single CPU thread. The 'LAN Interface' component stores network state, for talking to the Hub, and produces arrays of voltage values for processing. Voltage values are passed to the 'Spike Detection' component, which stores feedback values and spike counts, and passes recalibration commands back to the LAN Interface. When the pong environment is ready to run, it updates the state of the paddle based on the spike counts, updates the state of the ball based on its velocity and collision conditions, and reconfigures the stimulation sequencer based on the relative position of the ball and current state of the game. The stimulation sequencer stores and updates indices and countdowns relating to the stimulations it must produce and converts these into commands each time the corresponding countdown reaches zero, which are finally passed back to the LAN Interface, to send to the MEA system, closing the loop. The procedures associated with each component are run one after the other in a simple loop control flow, but the 'pong' environment only moves forward every 200th update, short-circuiting otherwise. Additionally, up to three worker processes are launched in parallel, depending on which parts of the system need to be recorded. They receive data from the main thread via shared memory and write it to file, allowing the main thread to continue processing data without having to hand control to the operating system and back again. **B)** Numeric operations in the real-time spike detection component of the *DishBrain* closed loop system, including multiple IIR filters. Running a virtual environment in a closed loop imposes strict performance requirements, and digital signal processing is the main bottleneck of this system, with close to 40 MiB of data to process every second. Simple sequences of IIR digital filters is applied to incoming data, storing multiple arrays of 1024 feedback values in between each sample. First, spikes on the incoming data are detected by applying a high pass filter to determine the deviation of the activity, and comparing that to the MAD, which is itself calculated with a subsequent low pass filter. Then, a low pass filter is applied to the original data to determine whether the MEA hardware needs to be recalibrated, affecting future samples. This system was able to keep up with the incoming data on a single thread of an Intel Core i7-8809G.

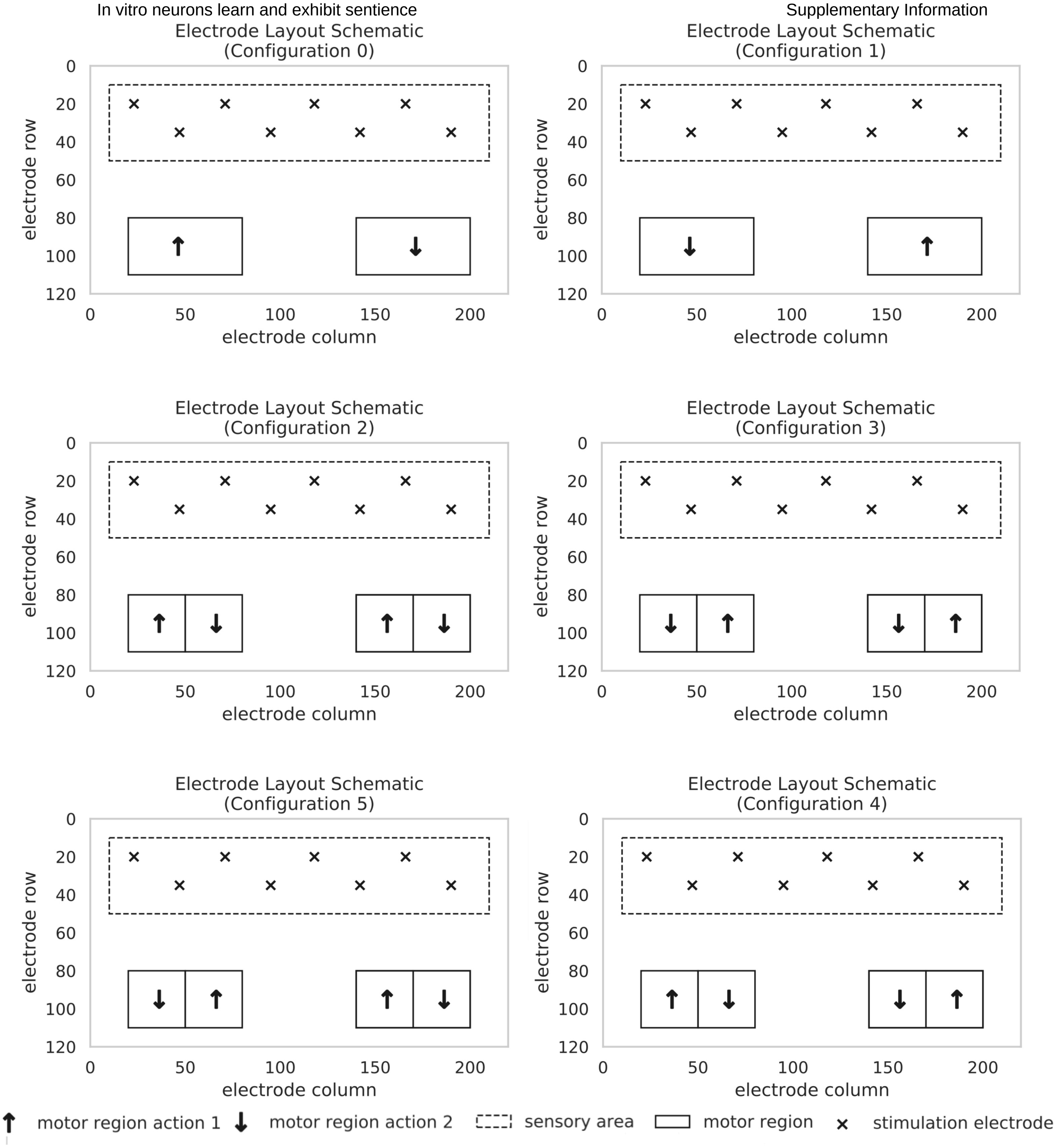

Fig. S3. Representation of the specific configurations of the DishBrain platform, related to Fig. 4E. Stimulation is delivered to a predefined sensory area and activity is measured in the motor regions to determine how the paddle will move. Feedback is provided via the sensory area based on the outcome of the motor region activity. Note the different configurations in which motor activity may have been interpreted. Configuration 0 was initially adopted as the beginning choice, however when the EXP3 algorithm was used to control selection from all of the above options, experimental cultures adopted a preference for configuration 3, which was then adopted going forward.

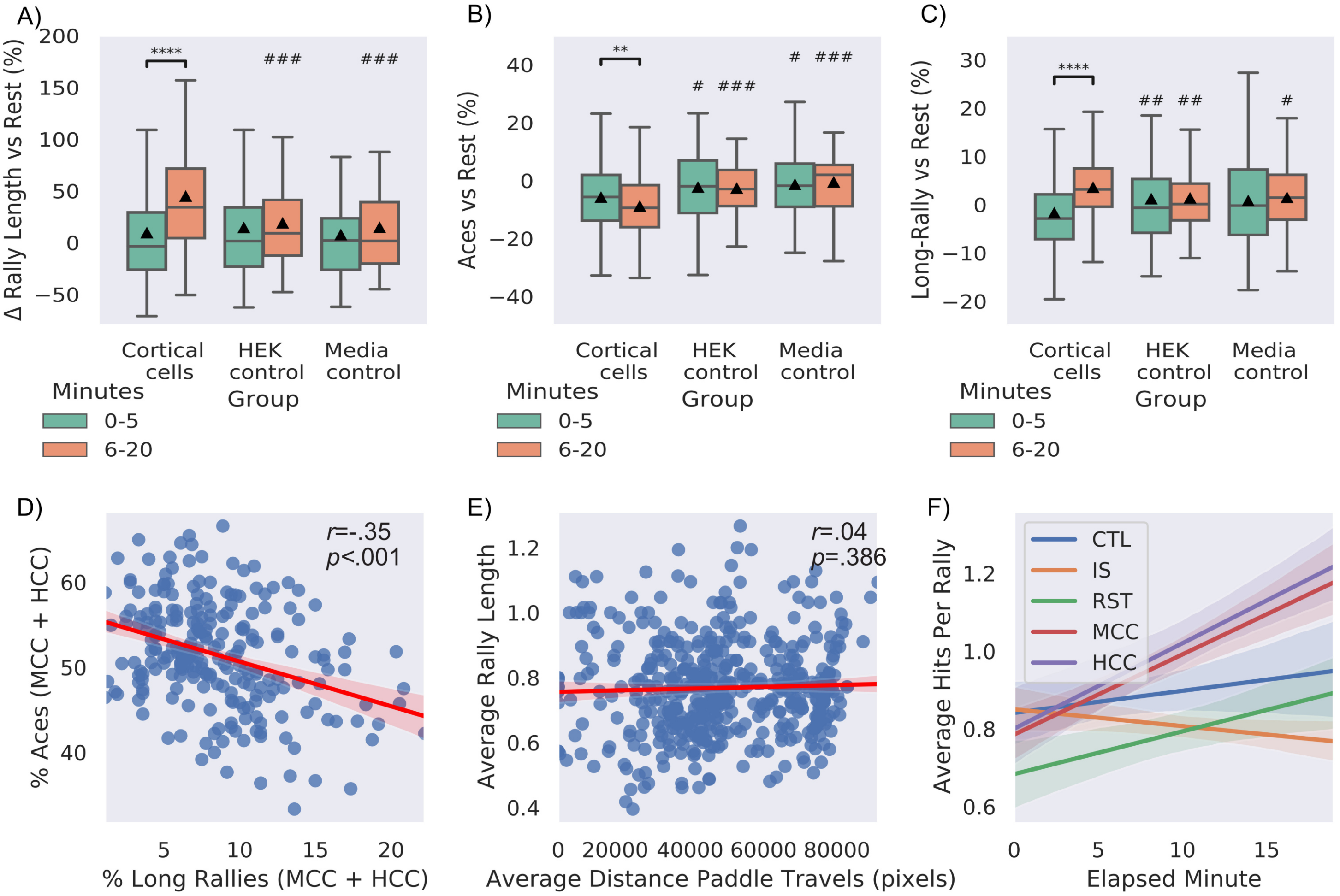

Fig. S4. Further controls and follow up for initially investigation into learning effects, related to Fig. 5. Significance bars show within group differences denoted with *. Symbols show between group differences at the given timepoint: # = vs Cortical cells. The number of symbols denotes the p-value cut off, where 1 = p < 0.05, 2 = p <0.01, 3 = p < 0.001 and 4 = p <0.0001. Box plots show interquartile range, with bars demonstrating 1.5X interquartile range, the line marks the median and ▲marks the mean. Electrically inactive non-neural cells also display no learning over time and perform at media control levels compared to cortical cells. **A)** Looking at the % change in rally length compared to match rest controls, cortical cells condition showed significant $t = 8.22$, p = 1.15^{-15}) and outperformed HEK293T cells and media control groups at timepoint 2 which showed no change over time (**Table S3**). **B)** Shows similar differences vs rest performance for aces across conditions, where the Cortical cell group showed significantly less % of aces across time ($t = 3.21$, p = 0.002) along with significantly fewer aces than the HEK control and Media control groups at both timepoints (**Table S3**). **C)** differences vs rest performance for % if long-rallies across conditions, where the Cortical cell group showed significantly more long-rallies across time ($t = 3.40$, p = 0.0007) along with significantly fewer aces than the HEK control and Media control groups at the second timepoint (**Table S3**). **D)** Significant negative correlation ($r = -0.35$, $p < 0.001$) between % aces and % long rallies for experimental cultures by session. **E)** No statistically significant correlation was observed between average paddle distance moved in a session and average rally length. This supports that paddle movement alone could explain the observed learning effects for neural cultures embodied in closed-loop feedback. **F)** Learning effects overtime when embodied in a closed-loop stimulation can also be observed with linear regression. After controlling for family wise error with a Bonferroni correction, a significant linear regression was found for both the human cortical cells (HCC) ($R^2 = 0.007$, $F(1, 2698) = 27.51$, $p = 0.0001$, $\beta = 0.30$, $p < 0.001$) and mouse cortical cells (MCC) ($R^2 = 0.015$, $F(1, 1875) = 28.06$, $p = 6.55^{-7}$, $\beta = 0.71$, $p < 0.001$). After correction, no significant linear regression was found for the rest condition (RST) ($R^2 = 0.006$, $F(1, 836) = 5.07$, $p = 0.123$, $\beta = 0.55$, p =0.125), media control (CTL) ($R^2 = 0.001$, $F(1, 1279) = 1.72$, $p = 0.950$, $\beta = 0.24$, p =0.950), or the in-silico control (IS) ($R^2 = 0.003$, $F(1, 758) = 2.49$, $p = 0.575$, $\beta = -0.76$, p =0.575). While these R values are relatively small due to the other factors explored throughout this paper, this is simply another way to showcase the learning pattern seen over time in cells but not in control conditions, suggesting that the increase in the average rally length is not chance alone.

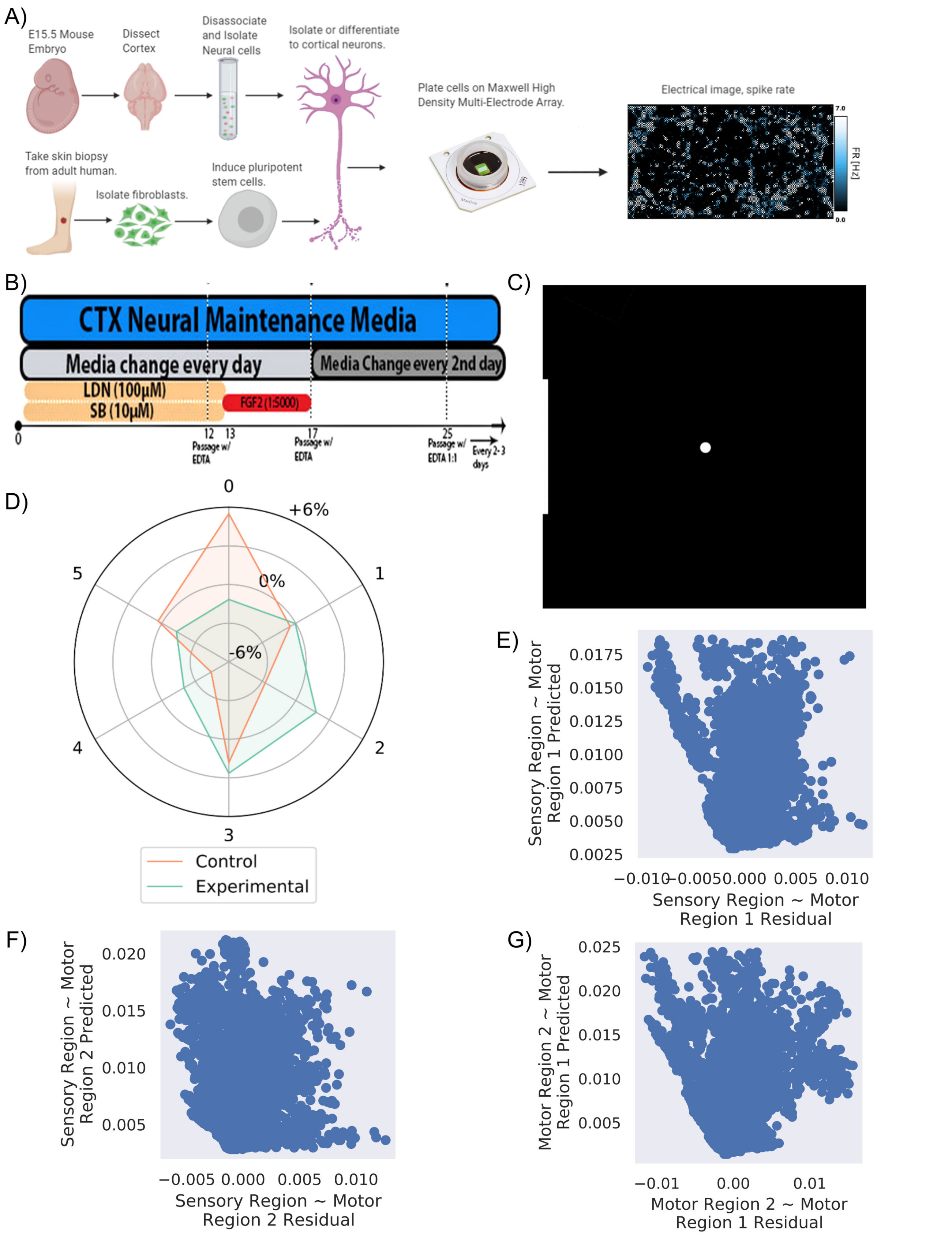

Fig. S5. Key methods used in this study, related to Star Methods. A) Diagrammatic illustration of the core experimental setup which drove the research in this project. **B)** Illustration of Dual SMAD inhibition protocol for differentiating pluripotent cells into cortical cells. **C)** Starting position of paddle and ball as visualised in the *DishBrain* platform. From the perspective of the neural cultures, it is more accurate to imagine that they view this world from the perspective of the paddle looking at the ball opposed to top-down as presented here. **D)** Shows the distribution differences relative to chance in percentage that a motor configuration was chosen by EXP3 algorithm ($\chi 2$ = 35690.93, p<0.0001) for control and experimental cultures. Motor configuration 0 was selected most often for media control while motor configuration 3 was selected most often for experimental. **E) – G)** The predicted vs residual values for the regression testing the assumption of linearity between variables: **E)** Motor Region 1 from Sensory Region, **F)** Motor Region 2 from Sensory region, and **G)** Motor Region 1 from Motor Region 2.

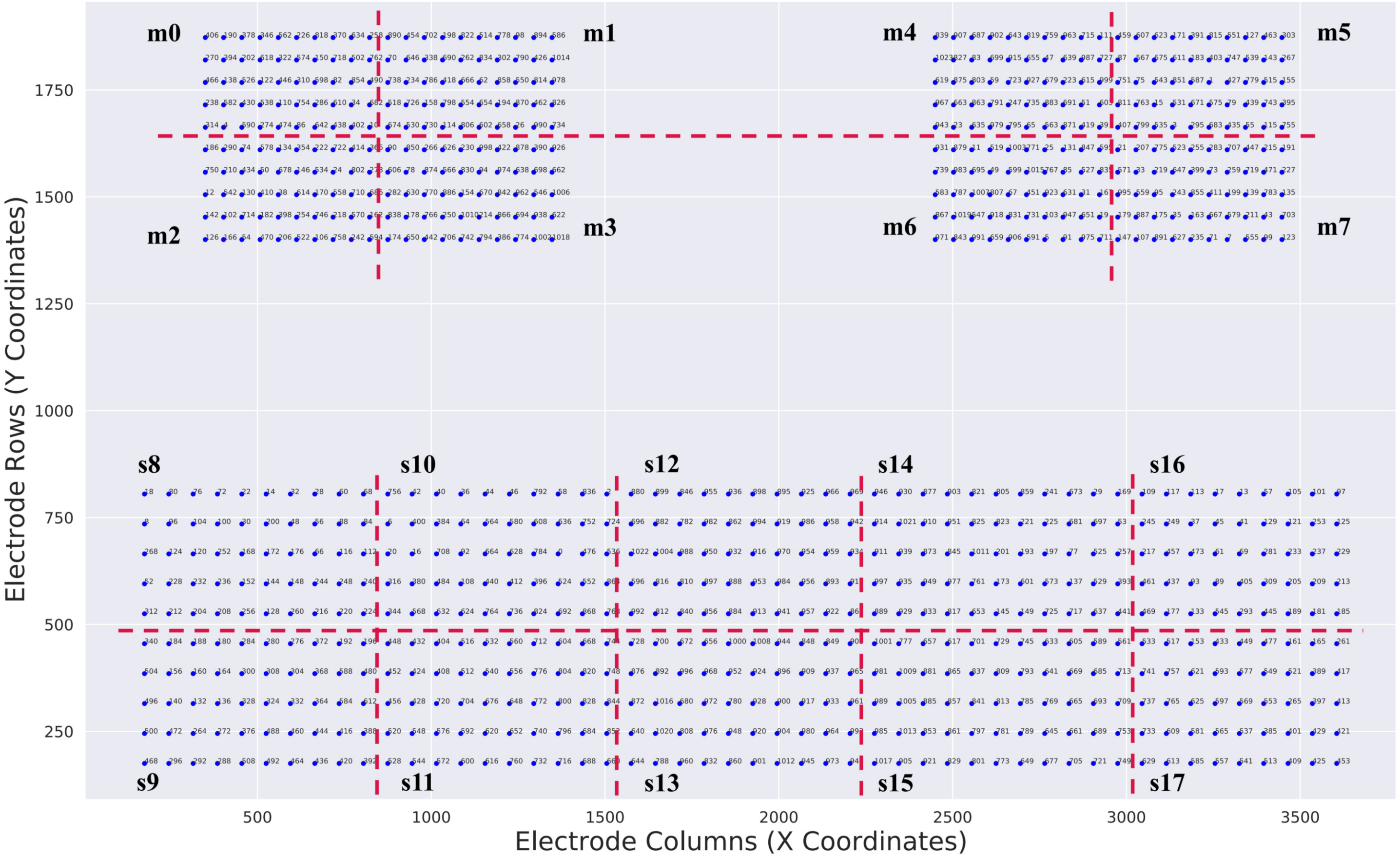

Fig. S6. Schematic of spatial clustering used to calculate subregions for mean local information entropy, related to Fig. 7 and Star Methods. The spatial clustering of channels is represented according to their x and y coordinates on the MEA surface. Each cluster contains 50 channels. Motor area groups are labelled with m_i while clusters of sensory channels are labelled by s_i

Table S1, related to Fig. 4, 5, 6, 7, S4: Multivariate statistical tests and all results for tests done, including figure panel, parameter assessed, statistical source and test, degrees of freedom, mean square and F values, p-value and partial eta square estimate of effect size (np^2).

Figure	Panel	Parameters	Source	DF1	DF2	MS	F	p-value	np^2	Method
**4**	E	Average Rally Length	Group - all	1	845	0.305	10.381	0.001	0.012	ANOVA
			Half - all	2	845	0.446	15.172	0.000	0.035	
			Interaction - all	2	845	0.078	2.646	0.072	0.006	
**5**	B	Average Rally Length	Group - all	4	394	0.297	3.330	0.011	0.033	RM ANOVA
			half - all	1	394	9.208	98.908	0.000	0.201	
			Interaction - all	4	394	2.020	21.696	0.000	0.181	
			Group – time 1	4	394	0.698	7.031	0.000	0.067	ANOVA
			Group – time 2	4	394	1.619	19.519	0.000	0.165	ANOVA
	C	% Aces	Group - all	4	394	0.081	9.284	0.000	0.086	RM ANOVA
			half - all	1	394	0.131	16.509	0.000	0.040	
			Interaction - all	4	394	0.058	7.295	0.000	0.069	
			Group – time 1	4	394	0.044	4.143	0.003	0.040	ANOVA
			Group – time 2	4	394	0.095	15.583	0.000	0.137	ANOVA
	D	% Long Rally	Group - all	4	394	0.017	4.767	0.001	0.046	RM ANOVA
			half - all	1	394	0.206	59.746	0.000	0.132	
			Interaction - all	4	394	0.047	13.531	0.000	0.121	
			Group – time 1	4	394	0.046	10.191	0.000	0.094	ANOVA
			Group – time 2	4	394	0.017	6.928	0.000	0.066	ANOVA
	E	Paddle Distance	Within	4	776	2.51^{10}	77.63	0.000	0.29	ANOVA
**6**	C	Average Rally Length	Group - all	2	353	20740	4.721	0.000	0.026	RM ANOVA
			half - all	1	353	33440	16.577	0.000	0.045	
			Interaction - all	2	353	25812	12.795	0.000	0.068	
			Group – time 1	2	483	7559	2.181	0.114	0.009	ANOVA
			Group – time 2	2	483	53943	20.507	0.000	0.078	ANOVA
	D	% Change Average Rally Length vs. Rest	Group - all	2	164	49314	7.674	0.001	0.086	RM ANOVA
			Test-day - all	2	328	16.115	0.037	0.963	0.000	
			Interaction - all	4	328	908.448	2.100	0.081	0.025	
	E	% Ace vs Rest	Group - all	2	353	19992	6.511	0.002	0.036	RM ANOVA
			half - all	1	353	42.70	0.646	0.422	0.002	
			Interaction - all	2	353	549.025	8.308	0.000	0.045	
			Group – time 1	2	483	453.464	2.181	0.127	0.008	ANOVA
			Group – time 2	2	483	2906	18.096	0.000	0.070	ANOVA
	F	% Ace vs Rest	Group - all	2	164	2683	12.125	0.000	0.129	RM ANOVA
			Test-day - all	2	328	110.546	0.971	0.380	0.006	
			Interaction - all	4	328	180.459	1.585	0.178	0.019	
	G	% Long-Rally vs Rest	Group - all	2	353	52.007	0.650	0.523	0.004	RM ANOVA
			half - all	1	353	1089.865	29.932	0.000	0.078	
			Interaction - all	2	353	436.936	12.000	0.000	0.064	

			Group – time 1	2	483	617.708	8.513	0.000	0.034	ANOVA
			Group – time 2	2	483	162.934	3.219	0.041	0.013	ANOVA
	H	% Long-Rally vs Rest	Group - all	2	164	154.446	0.490	0.614	0.006	RM ANOVA
			Test-day - all	2	328	21.678	-0.244	1.000	0.00	
			Interaction - all	4	328	118.779	-1.336	1.000	0.00	
**7**	J	Mean Information Entropy	Rest vs Gameplay	1	232	0.066	59.29	0.000	0.204	RM ANOVA
			Feedback	1	232	0.01	319.73	0.000	0.580	
			Interaction	1	232	0.001	40.44	0.000	0.148	
	K	Normalised Mean Information Entropy	Rest vs Gameplay	1	232	2.11^{-7}	29.438	0.000	0.113	RM ANOVA
			Feedback	1	232	2.67^{-6}	510.82	0.000	0.688	
			Interaction	1	232	2.95^{-7}	56.457	0.000	0.196	
	L	Normalised Mean Information Entropy	Condition	2	513	1.47^{-7}	4.315	0.000	0.016	RM ANOVA
			Feedback	1	513	4.65^{-6}	1174.7	0.000	0.696	
			Interaction	2	513	1.15^{-6}	291.11	0.000	0.532	
	I	Distance from the Mean Centre of Activity During Rest	Gameplay vs Rest	1	466	164159.557	191.949	0.000	0.292	RM ANOVA
**S4**	A	% Change Average Rally Length vs. Rest	Group - all	2	237	13897	3.052	0.049	0.02	RM ANOVA
			half - all	1	237	63857	27.008	0.000	0.102	
			Interaction - all	2	237	13814	5.843	0.003	0.047	
			Group – time 1	2	443	1389	0.405	0.667	0.002	ANOVA
			Group – time 2	2	442	42205	14.107	0.000	0.060	ANOVA
	B	% Ace vs Rest	Group - all	2	237	1956	10.036	0.000	0.078	RM ANOVA
			half - all	1	237	378	10.036	0.010	0.028	
			Interaction - all	2	237	258	4.596	0.011	0.037	
			Group – time 1	2	443	844	5.060	0.007	0.022	ANOVA
			Group – time 2	2	442	2828	26.297	0.000	0.106	ANOVA
	C	% Long-rallies vs Rest	Group - all	2	237	47.90	0.791	0.454	0.007	RM ANOVA
			half - all	1	237	1507	33.155	0.000	0.123	
			Interaction - all	2	237	344	7.585	0.001	0.060	
			Group – time 1	2	443	425	6.063	0.003	0.027	ANOVA
			Group – time 2	2	442	258.7	6.029	0.003	0.027	ANOVA

Table S2, related to Fig. 4, 5, 6, 7: Follow up main text post-hoc tests for multivariate tests, including means, standard error (SE), t-scores, degree of freedom and exact p-values with hedges.

Figure	Panel	Parameters	A	B	Mean A	Mean B	SE	T	df	p-value	Hedges	Method
**4**	E	Average Rally Length – Second Timepoint	MCC	HCC	0.801	0.843	0.012	-3.453	591.982	0.001	-0.254	Games-Howell Nonparametric Post-hoc Test
			Test 1	Test 2	0.780	0.823	0.019	-2.218	125.002	0.072	-0.240	
			Test 1	Test 3	0.780	0.901	0.025	-4.767	218.363	0.001	-0.637	
			Test 2	Test 3	0.823	0.901	0.019	-4.138	163.172	0.001	-0.402	
**5**	B	Average Rally Length – First Timepoint	CTL	HCC	0.9	0.674	0.05	4.513	153.939	0.001	0.632	Games-Howell Nonparametric Post-hoc Test
			CTL	IS	0.9	0.832	0.052	1.298	113.864	0.67	0.254	
			CTL	MCC	0.9	0.78	0.051	2.359	151.218	0.132	0.352	
			CTL	RST	0.9	0.765	0.055	2.451	114.874	0.109	0.464	
			HCC	IS	0.674	0.832	0.043	-3.66	101.344	0.004	-0.668	
			HCC	MCC	0.674	0.78	0.041	-2.568	228.111	0.08	-0.335	
			HCC	RST	0.674	0.765	0.047	-1.967	96.31	0.29	-0.345	
			IS	MCC	0.832	0.78	0.044	1.18	100.059	0.736	0.223	
			IS	RST	0.832	0.765	0.049	1.372	77.617	0.629	0.304	
			MCC	RST	0.78	0.765	0.047	0.317	96.415	0.9	0.058	
		Average Rally Length – Second Timepoint	CTL	HCC	0.872	1.129	0.043	-5.919	195.83	0.001	-0.829	Games-Howell Nonparametric Post-hoc Test
			CTL	IS	0.872	0.801	0.037	1.928	114.885	0.309	0.377	
			CTL	MCC	0.872	1.02	0.04	-3.667	161.682	0.003	-0.547	
			CTL	RST	0.872	0.815	0.049	1.154	93.05	0.751	0.219	
			HCC	IS	1.129	0.801	0.036	9.189	168.072	0.001	1.676	
			HCC	MCC	1.129	1.02	0.039	2.776	236.963	0.046	0.362	
			HCC	RST	1.129	0.815	0.048	6.467	96.724	0.001	1.135	
			IS	MCC	0.801	1.02	0.032	-6.872	131.12	0.001	-1.301	
			IS	RST	0.801	0.815	0.043	-0.325	60.15	0.9	-0.072	
			MCC	RST	1.02	0.815	0.046	4.472	79.017	0.001	0.817	
			CTL	HCC	0.872	1.129	0.043	-5.919	195.83	0.001	-0.829	
	C	% Aces – First Timepoint	CTL	HCC	0.508	0.545	0.016	-2.364	145.517	0.131	-0.331	Games-Howell Nonparametric Post-hoc Test
			CTL	IS	0.508	0.535	0.019	-1.44	99.395	0.59	-0.282	
			CTL	MCC	0.508	0.534	0.017	-1.52	163.66	0.544	-0.227	
			CTL	RST	0.508	0.585	0.019	-4.174	106.983	0.001	-0.79	
			HCC	IS	0.545	0.535	0.016	0.634	70.432	0.9	0.116	
			HCC	MCC	0.545	0.534	0.014	0.806	205.214	0.9	0.105	
			HCC	RST	0.545	0.585	0.016	-2.587	78.04	0.083	-0.454	
			IS	MCC	0.535	0.534	0.017	0.063	87.795	0.9	0.012	
			IS	RST	0.535	0.585	0.019	-2.705	77.755	0.062	-0.6	
			MCC	RST	0.534	0.585	0.017	-3.033	96.296	0.025	-0.554	
			CTL	HCC	0.53	0.482	0.012	3.956	124.102	0.001	0.554	

		% Aces – Second Timepoint	CTL	IS	0.53	0.556	0.013	-2.017	115.384	0.264	-0.395	Games-Howell Nonparametric Post-hoc Test
			CTL	MCC	0.53	0.499	0.013	2.367	150.917	0.13	0.353	
			CTL	RST	0.53	0.57	0.019	-2.147	79.955	0.211	-0.407	
			HCC	IS	0.482	0.556	0.009	-8.323	94.994	0.001	-1.518	
			HCC	MCC	0.482	0.499	0.01	-1.731	195.047	0.419	-0.226	
			HCC	RST	0.482	0.57	0.016	-5.352	52.462	0.001	-0.939	
			IS	MCC	0.556	0.499	0.01	5.523	121.048	0.001	1.045	
			IS	RST	0.556	0.57	0.017	-0.882	55.815	0.9	-0.196	
			MCC	RST	0.499	0.57	0.017	-4.138	62.492	0.001	-0.756	
	D	% Long Rally – First Timepoint	CTL	HCC	0.095	0.044	0.011	4.598	117.248	0.001	0.644	Games-Howell Nonparametric Post-hoc Test
			CTL	IS	0.095	0.093	0.013	0.136	107.875	0.9	0.027	
			CTL	MCC	0.095	0.073	0.012	1.791	148.918	0.384	0.267	
			CTL	RST	0.095	0.092	0.012	0.235	120	0.9	0.045	
			HCC	IS	0.044	0.093	0.01	-4.824	60.589	0.001	-0.88	
			HCC	MCC	0.044	0.073	0.009	-3.387	186.286	0.008	-0.442	
			HCC	RST	0.044	0.092	0.009	-5.554	82.198	0.001	-0.975	
			IS	MCC	0.093	0.073	0.011	1.758	85.357	0.407	0.333	
			IS	RST	0.093	0.092	0.012	0.093	72.665	0.9	0.021	
			MCC	RST	0.073	0.092	0.01	-1.886	115.124	0.331	-0.344	
		% Long Rally – Second Timepoint	CTL	HCC	0.093	0.106	0.008	-1.641	118.934	0.475	-0.23	Games-Howell Nonparametric Post-hoc Test
			CTL	IS	0.093	0.083	0.008	1.154	110.96	0.751	0.226	
			CTL	MCC	0.093	0.122	0.009	-3.205	148.722	0.014	-0.478	
			CTL	RST	0.093	0.087	0.011	0.515	102.083	0.9	0.098	
			HCC	IS	0.106	0.083	0.005	4.313	108.408	0.001	0.787	
			HCC	MCC	0.106	0.122	0.006	-2.416	189.523	0.116	-0.315	
			HCC	RST	0.106	0.087	0.009	2.173	59.384	0.204	0.381	
			IS	MCC	0.083	0.122	0.006	-5.911	131.734	0.001	-1.119	
			IS	RST	0.083	0.087	0.009	-0.453	59.221	0.9	-0.1	
			MCC	RST	0.122	0.087	0.009	3.619	77.747	0.005	0.661	
	E	Paddle Distance	CTL	HCC	40634.11	-11366.3	2259.891	-5.02959	325.7946	0.001	40634.11	Games-Howell Nonparametric Post-hoc Test
			CTL	IS	40634.11	-37450.8	1771.768	-21.1375	153.6698	0.001	40634.11	
			CTL	MCC	40634.11	-9373.39	1998.548	-4.6901	216.7304	0.001	40634.11	
			CTL	RST	40634.11	7233.647	1912.057	3.78317	203.6913	0.00189	40634.11	
			HCC	IS	52000.43	-26084.5	1476.495	-17.6665	289.6122	0.001	52000.43	
			HCC	MCC	52000.43	1992.923	1742.146	1.14395	355.7488	0.7562	52000.43	
			HCC	RST	52000.43	18599.96	1642.206	11.3262	406.2486	0.001	52000.43	
			IS	MCC	78084.88	28077.38	1033.018	27.17995	114.3084	0.001	78084.88	
			IS	RST	78084.88	44684.42	853.7542	52.33874	269.532	0.001	78084.88	
			MCC	RST	50007.5	16607.04	1258.542	13.19546	219.0208	0.001	50007.5	

**6**	C	% Change Average Rally Length vs. Rest Second Timepoint	STIM	SIL	43.351	21.394	6.006	3.656	309.729	0.001	0.396	Games-Howell Nonparametric Post-hoc Test
			STIM	NF	43.351	8.175	4.604	7.64	339.144	0.001	0.854	
			SIL	NF	21.394	8.175	5.091	2.596	198.782	0.027	0.324	
	D	% Change Average Rally Length vs. Rest Second Timepoint – Test Day	STIM	SIL	43.351	21.394	6.006	3.656	309.729	0.001	0.396	Games-Howell Nonparametric Post-hoc Test
			STIM	NF	43.351	8.175	4.604	7.64	339.144	0.001	0.854	
			SIL	NF	21.394	8.175	5.091	2.596	198.782	0.027	0.324	
	E	% Ace vs. Rest First Timepoint	STIM	SIL	-5.633	-4.809	1.502	-0.548	267.191	0.831	-0.059	Games-Howell Nonparametric Post-hoc Test
			STIM	NF	-5.633	-2.283	1.835	-1.826	195.485	0.164	-0.204	
			SIL	NF	-4.809	-2.283	2.016	-1.253	230.34	0.425	-0.156	
		% Ace vs. Rest Second Timepoint	STIM	SIL	-8.669	-1.871	-6.798	-5.795	288.862	0.001	-0.628	Games-Howell Nonparametric Post-hoc Test
			STIM	NF	-8.669	-1.606	-7.063	-4.174	178.239	0.001	-0.466	
			SIL	NF	-1.871	-1.606	-0.265	-0.149	200.322	0.9	-0.019	
	F	% Ace vs. Rest Second Timepoint - Test Day	STIM	SIL	-8.669	-1.871	-6.798	-5.795	288.862	0.001	-0.628	Games-Howell Nonparametric Post-hoc Test
			STIM	NF	-8.669	-1.606	-7.063	-4.174	178.239	0.001	-0.466	
			SIL	NF	-1.871	-1.606	-0.265	-0.149	200.322	0.9	-0.019	
	G	%Long rally vs. Rest First Timepoint	STIM	SIL	-1.689	1.8	0.959	-3.64	234.35	0.001	-0.394	Games-Howell Nonparametric Post-hoc Test
			STIM	NF	-1.689	1.082	0.957	-2.896	215.082	0.012	-0.323	
			SIL	NF	1.8	1.082	1.152	0.623	253.482	0.788	0.078	
		%Long rally vs. Rest Second Timepoint	STIM	SIL	3.48	2.195	0.794	1.619	250.019	0.24	0.176	Games-Howell Nonparametric Post-hoc Test
			STIM	NF	3.48	1.58	0.794	2.393	229.45	0.046	0.267	
			SIL	NF	2.195	1.58	0.933	0.659	253.41	0.767	0.082	
	H	%Long rally vs. Rest Second Timepoint - Test Day	STIM	SIL	3.48	2.195	0.794	1.619	250.019	0.24	0.176	Games-Howell Nonparametric Post-hoc Test
			STIM	NF	3.48	1.58	0.794	2.393	229.45	0.046	0.267	
			SIL	NF	2.195	1.58	0.933	0.659	253.41	0.767	0.082	
**7**	L	Normalized Mean Information Entropy – Prior to Feedback	NF	STIM	3.65^{-3}	3.02^{-3}	0.12^{-3}	5.333	309.855	0.001	0.588	Games-Howell Nonparametric Post-hoc Test
			NF	SIL	3.65^{-3}	2.81^{-3}	0.11^{-3}	7.627	241.122	0.001	0.934	
			STIM	SIL	3.02^{-3}	2.81^{-3}	0.11^{-3}	1.984	387.645	0.118	0.207	
		Normalized Mean Information Entropy – Post Feedback	NF	STIM	3.46^{-3}	4.49^{-3}	0.15^{-3}	-6.734	367.936	0.001	-0.742	Games-Howell Nonparametric Post-hoc Test
			NF	SIL	3.46^{-3}	5.23^{-3}	0.13^{-3}	-13.411	262.573	0.001	-0.1643	
			STIM	SIL	4.49^{-3}	5.23^{-3}	0.16^{-3}	-4.511	391.431	0.001	-0.470	

Table S3, related to Fig. 5: Follow up post-hoc tests performed on data shown in **Fig. S4** for multivariate tests, including means, standard error (SE), t-scores, degree of freedom and exact p-values with hedges.

Figure	Panel	Parameters	A	B	Mean A	Mean B	SE	T	df	p-value	Hedges	Method
**S4**	A	% Change Average Rally Length vs. Rest Second Timepoint	CCs	HEK	44.583	18.682	5.825	4.446	279.739	0.001	0.493	Games-Howell Nonparametric Post-hoc Test
			CCs	Media	44.583	14.374	6.652	4.541	153.166	0.001	0.591	
			HEK	Media	18.682	14.374	7.074	0.609	163.673	0.796	0.089	
	B	% Ace vs. Rest First Timepoint	CCs	HEK	-5.888	-2.41	1.43	-2.433	241.772	0.041	-0.27	Games-Howell Nonparametric Post-hoc Test
			CCs	Media	-5.888	-1.422	1.649	-2.709	132.14	0.021	-0.352	
			HEK	Media	-2.41	-1.422	1.837	-0.538	164.491	0.837	-0.078	
		% Ace vs. Rest Second Timepoint	CCs	HEK	-8.953	-2.741	1.054	-5.894	292.584	0.001	-0.654	Games-Howell Nonparametric Post-hoc Test
			CCs	Media	-8.953	-0.617	1.405	-5.931	129.919	0.001	-0.772	
			HEK	Media	-2.741	-0.617	1.452	-1.463	137.53	0.313	-0.213	
	C	%Long rally vs. Rest Second Timepoint	CCs	HEK	-1.767	1.153	0.963	-3.033	200.551	0.008	-0.336	Games-Howell Nonparametric Post-hoc Test
			CCs	Media	-1.767	0.765	1.184	-2.139	107.676	0.087	-0.278	
			HEK	Media	1.153	0.765	1.366	0.284	157.623	0.9	0.041	
		%Long rally vs. Rest Second Timepoint - Test Day	CCs	HEK	3.523	1.309	0.734	3.016	236.704	0.008	0.335	Games-Howell Nonparametric Post-hoc Test
			CCs	Media	3.523	1.424	0.824	2.548	134.013	0.032	0.332	
			HEK	Media	1.309	1.424	0.929	-0.124	169.022	0.9	-0.018	

Table S4, related to STAR methods: Percentage configurations selected (in bold) by EXP3 algorithm for control and experimental groups during pilot testing as shown in **Fig. S5D**.

Configuration	Control %	Experimental %
**0**	22.17	15.51
**1**	16.16	16.62
**2**	13.93	18.50
**3**	18.49	19.31
**4**	12.25	14.69
**5**	17.01	15.37
